# Hyperplasia of Interstitial Cells of Cajal in Sprouty Homolog 4 Deficient Mice

**DOI:** 10.1371/journal.pone.0124861

**Published:** 2015-04-29

**Authors:** An Thys, Pierre Vandenberghe, Perrine Hague, Ophir D. Klein, Christophe Erneux, Jean-Marie Vanderwinden

**Affiliations:** 1 Laboratory of Neurophysiology, Faculty of Medicine, Université Libre de Bruxelles, Brussels, Belgium; 2 Department of Orofacial Sciences and Program in Craniofacial and Mesenchymal Biology, University of California, San Francisco, California, United States of America; 3 Department of Pediatrics and Institute for Human genetics, University of California, San Francisco, California, United States of America; 4 IRIBHM, Faculty of Medicine, Université Libre de Bruxelles, Brussels, Belgium; University of Pittsburgh Cancer Institute, UNITED STATES

## Abstract

Gastrointestinal stromal tumors, which are thought to derive from interstitial cells of Cajal or their precursors, often harbor an oncogenic mutation of the KIT receptor tyrosine kinase. Sprouty homolog 4, a known negative regulator of ERK pathway, has been identified in the interstitial cells of Cajal in the *Kit^K641E^* murine model of gastrointestinal stromal tumors. Sprouty homolog 4 was upregulated both at the mRNA and protein level in these cells, suggesting that Sprouty homolog 4 is downstream of oncogenic KIT activation and potentially engaged in the negative feedback loop of ERK activation in this model. Here, we used *Kit^K641E^* heterozygous and *Sprouty homolog 4* knock out animals to quantify interstitial cells of Cajal *in situ*, using quantitative immunofluorescence for the receptor tyrosine kinase Kit and for phosphodiesterase 3a (PDE3A). In the antrum of *Sprouty homolog 4* knock out mice, hyperplasia of interstitial cells of Cajal was reminiscent of the *Kit^K641E^* heterozygous mice antrum. Additionally, the density of interstitial cells of Cajal was higher in the colon of adult *Sprouty homolog 4* knock out mice than in WT littermates, although hyperplasia seemed more severe in *Kit^K641E^* heterozygous mice. Functional transit studies also show similarities between *Sprouty homolog 4* knock out and *Kit^K641E^* heterozygous mice, as the total transit time in 9 month old animals was significantly increased in both genotypes compared to WT littermates. We concluded that the lack of *Sprouty homolog 4* expression leads to hyperplasia of the interstitial cells of Cajal and is functionally associated with a delayed transit time.

## Introduction

Gastrointestinal stromal tumors (GIST) are the most common sarcoma of the gastrointestinal tract. They are thought to derive from the interstitial cells of Cajal (ICC), or an ICC precursor, and are highly resistant to conventional chemotherapy and radiotherapy [1,2]. Approximately 85% of GIST harbor oncogenic KIT mutations [3]. KIT (a.k.a. c-kit) belongs to the family of receptor tyrosine kinases (RTK) and is a well-established ICC marker [4].

RTK play central roles in multiple biological processes such as proliferation, survival, differentiation and migration [5]. Activation of KIT by its ligand, Stem Cell Factor (SCF), is required for the development of ICC, hematopoietic stem cells, melanocytes, mast cells and germ cells. A number of downstream signaling cascades are activated by the SCF/KIT pathway, including RAS/ERK, PI3K/AKT/mTOR, JAK/STAT and PLC pathway [6,7]. Aberrant activation of these KIT signaling pathway has been linked to various human cancers, including GIST, as well as with developmental disorders [3]. Under normal circumstances, RTK signaling pathways are tightly controlled by negative-feedback loops, such as those mediated by Sprouty homolog proteins (SPRYs), in order to prevent excessive signaling that could lead to abnormal cellular behavior and disease [8].

Imatinib mesylate (Gleevec, STI571), a pharmacological inhibitor of KIT, has proven to be effective in patients with GIST. However, resistance often emerges and remains a major concern [9–11]. Hence, further studies of the signaling pathways downstream of KIT and its oncogenic mutant forms are important for further development of innovative targeted therapies.

Sprouty genes encode highly conserved negative feedback modulators of growth factor-mediated extracellular signal-regulated kinase (ERK) activation [12]. They were originally identified in Drosophila (dSprouty) as a feedback inhibitor of fibroblast growth factor (FGF) signaling during tracheal branching [13]. In mammals, there are four Sprouty isoforms (SPRY1-4), which are expressed in an ERK-dependent manner. SPRY modulation of ERK activation can be induced by various stimuli, including EGF, FGF, VEGF, PDGF, and SCF [14]. Inappropriate activation of the Ras/Erk pathway has been reported in various human cancers, including GIST [15–18].

We have previously reported an increase of both *Spry4* mRNA and SPRY4 immunoreactivity (-ir) in *Kit* ^*K641E*^ homozygous (*Kit* ^*K641E/K641E*^) mice [19], a mouse model of human GIST harboring a K-to-E substitution of the KIT receptor at amino acid 642 [20–22]. These mice had a pronounced hyperplasia of ICC in the antrum [22]. Stable expression of KIT^K641E^ in BaF3 cells showed that this receptor is constitutively tyrosine phosphorylated [20] and leads to an upregulation of pERK and pAKT [23]. *Spry1* and *Spry4* were among the genes upregulated identified by microarray analysis in human GIST patients [24]. Furthermore, *Spry4* was one of the most significant downregulated imatinib-responsive genes [25]. This implies that SPRY4 lies downstream of KIT activation and is potentially engaged in the negative feedback loop of ERK activation in ICC.

Therefore, we hypothesized that loss of SPRY4 could cause increased phosphorylation of ERK upon KIT RTK activation, leading to hyperplasia of the ICC in antrum, and possibly in other parts of the gut. Hence, we investigated the role of SPRY4 in the ICC *in vivo* using a *Spry4* knockout (KO) mouse model in which the open reading frame of *Spry4* has been removed. In this model, abnormal dental development has previously been reported [26]. In another *Spry4* KO model, in which exon 2 was removed, growth retardation and polysyndactyly were described [27]. To the best of our knowledge, *Spry4* KO mice have not been used so far to study the influence of SPRY4 deficiency in ICC. *Kit* ^*K641E*^ heterozygous (*Kit* ^*WT/K64*1E^ mice) were used to compare the phenotype between *Spry4* KO mice and oncogenic KIT mutant mice. We show that *Spry4* deficiency leads to hyperplasia of the ICC in antrum, which is reminiscent to the phenotype of the Kit^WT/K641E^ mice. Furthermore, *Spry4* KO mice also showed an increase of ICC in colon. Functional transit studies showed that the total transit time was significantly increased both in *Spry4* KO and in Kit^K641E^ heterozygous mice.

## Materials and Methods

### Ethics statement

Study of Kit^K641E^ and *Spry4* KO mice was approved by the ethics committee for animal well-being of the Faculty of Medicine, Université Libre de Bruxelles (Protocol number 491N).

### Animals

Generation of *Spry4* KO and *Kit* ^*K641E*^ animals have been described by Klein et al. 2006 [26] and Rubin et al. 2005 [22], respectively. In our colony, *Kit* ^*K641E*/K641E^ and *Kit* ^*K641E/K641E*^ *-Spry4* KO mice died around 12 days after birth. Therefore, only WT, Kit^WT/K641E^ and *Spry4* KO genotypes could be investigated in adult (3 and 9 month old) mice. Genotyping was performed as described [22,26], with primers listed in Table 1. Body weight was determined before mice were sacrificed by cervical dislocation and decapitation. Small intestine, colon and stomach were promptly removed. The gastric antrum was delineated from corpus based on visual landmarks on the serosa. Luminal content was gently emptied and surrounding tissues (e.g. mesenteric fat) were carefully removed by sharp dissection without damaging the serosa.

**Table 1:** Primers used for genotyping.

Primers for genotyping	
***SPRY4 (Flox and KO)***	
**Sense**	CAG GAC TTG GGA GTG CTT CCT TAG
**Antisense (Flox)**	CCT AGT ACC TTT TTG GGG AGA G
**Antisense (KO)**	TAC AGC AGG AAT GGC TAC GGT G
***Kit*** ^***K641E***^	
**Sense**	AGT TGG CAG GGT TAG CAG AA
**Antisense**	AGA CTC ACC TCC CAC CGT

### Real time quantitative PCR (qPCR)

A minimum of three different RNA samples from P10 WT, *Kit* ^*K641E/K641E*^ and *Spry4* KO mice antrum were used. Total RNA was extracted using RNeasy MiniKit (Qiagen, Valencia, CA, USA) according to manufacturer’s instructions. Genomic DNA was removed using the RNase-Free DNase set (Qiagen). RNA was reverse transcribed with 200 units of M-MLV Reverse Transcriptase (Invitrogen, Eugene, Oregon, USA) in a reaction containing 1μg of random primers (Amersham Bioscience, Piscataway, NJ, USA), 10mM each dNTP, 1x First-Strand buffer and 100mM dithiothreitol followed by heat deactivation. The cDNA reverse transcription product was amplified with specific primers (Table 2) by qPCR using SYBR Green chemistry on a 7500 Real-time PCR system (Applied Biosystems, Foster City, CA, USA). Identical thermal profile conditions, namely 95°C for 10min, then 40 cycles of 95°C for 15sec and 60°C for 1min were used for all primer sets. Emitted fluorescence was measured during annealing/extension phase and amplification plots were generated using the Sequence Detection System. Transcriptional quantification relative to GAPDH and β-actin reference genes was performed using qBase+ software (Biogazelle, Zwijnaarde, Belgium).

**Table 2:** Primers used for qPCR.

Primers	Sequence
**GAPDH Fw**	TGTGTCCGTCGTGGATCTGA
**GAPDH Rev**	CCTGCTTCACCACCTTCTTGA
**B-Actin Fw**	AACCGTGAAAAGATGACCCAGAT
**B-Actin Rev**	GCCTGGATGGCTACGTACATG
**SPRY4 Fw**	TGACTCTGCAGCTCCTCAAAGA
**SPRY4 Rev**	TCACAGGGACGCTGCTCTG
**SPRY1 Fw**	CCAGATGATTGCTAACAAGGTCAG
**SPRY1 Rev**	GTGAATGAAACEACCATGACCTACAT
**SPRY2 Fw**	AAAGCCGCGATCACGGA
**SPRY2 Rev**	GGCTGCGACCCGTTGC
**SPRED1 Fw**	GGAGACGGCGACTTCTGACA
**SPRED1 Rev**	GACAGTGACGCTGCTCAGTCC

### Immunofluorescence (IF)

Tissues were fixed for 24h in fresh 4% paraformaldehyde, pH 7.4, and cryopreserved in sucrose solutions (10%, 20%, 30% w/v in water), overnight (o/n) each, embedded in OCT (Sakura Finetec Europe, Leiden, the Netherlands) and frozen at -80°C. Circumferential sections (16μm thick) were cut on a CM 3050S cryostat (Leica Microsystems GmbH, Wetzlar, Germany), collected on Superfrost Plus glass slides (Thermo Scientific, Waltham, MA, USA) and stored at -20°C until use.

IF was carried out as described [28]. Briefly, slides were brought to room temperature (RT), permeabilized and blocked for 1 hour in 10mM TBS pH 8.2 containing 0.1% Triton X-100 (Sigma, Saint Louis, MO, USA) and 10% normal horse serum (NHS). Primary antibodies were diluted in a TBS-Triton X-100 0.1% and 1% NHS solution and incubated overnight at RT in a humid chamber. Slides were washed in TBS and incubated at RT for 1 hour in TBS containing the secondary antibodies. Slides were washed and mounted using Glycergel (Dako, Glostrup, Denmark) + 2.5% DABCO (Sigma). SPRY4 antibody specificity was assessed by preabsorption with the antigenic peptide. In short, SPRY4 antibody was incubated in the presence of 0.1μg/ml or 1μg/ml SPRY4 immunogenic peptide (Santa Cruz Biotechnology, Inc., Dallas, TX, USA, sc-18607P) for 30 min at 4°C. Subsequently, IF was carried out as described above.

Primary and secondary antibodies used for IF are summarized in Table 3.

**Table 3:** Primary and secondary antibodies used for immunofluorescence.

**Primary antibodies**	**Supplier**	**Cat.N°**	**Host**	**Dilution**
***PDE3A*** [58]	MRC-PPU Reagents Dundee University, Nethergate, Dundee, United Kingdom	S721A	Sheep	1/1000
***SPRY4***	Santa Cruz technology	Sc-18607	Goat	1/500
***SPRY2***	Upstate Chicago, IL, USA	07–524	Rabbit	1/2000
***pERK***	Cell signaling technology, Danvers, MA, USA	9101	Rabbit	1/500
***pAKT (Ser473) XP***	Cell signaling technology	4060	Rabbit	1/50
***HuC/D***	Invitrogen	A21272	Mouse	1/100
***KIT***	DAKO	A4502	Rabbit	1/500
***KIT (M14)***	Santa Cruz technology, Santa Cruz, CA, USA	sc-1494	Goat	1:100
***pSTAT5a/b (Tyr694)***	Santa Cruz technology	Sc-101806	Rabbit	1/200
***P70S6***	Cell signaling technology	9205	Rabbit	1/200
**Secondary antibodies**	**Supplier**	**Cat.N°**	**Host**	**Dilution**
***Anti Rabbit Alexa 594***	Jackson Immunoresearch laboratories, Inc., West Grove, PA, USA	711-585-152	Donkey	1/200
***Anti Biotin DL549***	Jackson Immunoresearch laboratories, Inc.	200-502-211	Donkey	1/200
***Anti Sheep Alexa 488***	Jackson Immunoresearch laboratories, Inc.	713-545-147	Donkey	1/200
***Anti Goat Alexa 594***	Jackson Immunoresearch laboratories, Inc.	705-585-147	Donkey	1/200
***Anti Rabbit Alexa 488***	Jackson Immunoresearch laboratories, Inc.	711-545-125	Donkey	1/200

Slides were observed and imaged on an AxioImager Z1 fluorescent microscope (Zeiss, Jena, Germany), using a Plan Apochromat 20x/0.8 or EC Plan NeoFluar 40x/0.75 objective. Excitation was provided by a HBO 105W lamp. Band pass filters sets #49, #38; and #43 (Zeiss) were used to detect blue, green and red fluorochromes respectively. Images (1388 by 1040 pixels, pixel size (x-y): 0.32 micron by 0.32 micron) were acquired sequentially with an AxioCamMRm camera (Zeiss) as 3 x 12 bit RBG proprietary *.zvi files. Files were processed with AxioVision (4.6) software (Zeiss). Images were displayed in linear mode with manual contrast adjustment and exported as uncompressed. TIF files. Figures were prepared with Adobe Illustrator.

Quantification of PDE3A-ir, KIT-ir and HuC/D-ir was performed using the Fiji software [29]. The plugin “Stich grid of images” [30] was used to assemble images covering the entire circumference of the sample. The boundaries of the muscularis propria were delineated to extract the region of interest (ROI). The area of immunoreactivity for the ICC or neural body markers, KIT-ir and PDE3A-ir or HuC/D-ir, respectively, was determined by thresholding within the ROI of the muscularis propria.

### Confocal microscopy

High resolution confocal imaging was performed using a Zeiss LSM780 system fitted on an Observer Z1 inverted microscope equipped with a LD LCI C-Apochromat 40x/1.1 W objective (Zeiss). The 488 nm excitation wavelength of the Argon/2 laser, a main dichroic HFT 488 and a band-pass emission filter (BP500-550 nm) were used for selective detection of the green fluorochrome. The 543nm excitation wavelength of the HeNe1 laser, a main dichroic HFT 488/543/633 and a long-pass emission filter (BP565-605 nm) were used for selective detection of the red fluorochrome. A 405 nm blue diode, a main dichroic HFT 405 and a band-pass emission filter (BP435-485 nm) were used for selective detection of the DNA counterstain.

Z-stacks of images were acquired sequentially with a zoom factor of 2 and optimal (1 Airy unit) pinhole (scaling (x-y-z): 0.21 x 0.21 x 0.53 micron) and stored as 8-bit proprietary *.czi files. Single plane images were displayed using Zen2010 software (Zeiss) and exported as 8 bits uncompressed *.TIF images. Figures were prepared as above.

### Total gastrointestinal transit time

Total gastrointestinal transit time was carried out as described [31]. Briefly, 200μl of 6% (w/v) carmine red (Sigma) suspended in 0.5% methylcellulose (Sigma) was administered by gavage through a 20 gauge round-tip feeding needle (n = 4–11 in each genotype). The time at which gavage took place was recorded as T_0_. After gavage, fecal pellets were monitored for the presence of carmine red at 10 min intervals. The interval between T_0_ and the time of first observance of carmine red in stool was considered as total gastrointestinal transit time.

### Statistics

All data represent the mean ± SD. Statistical analysis was performed with Prism 6 software (GraphPad Software, Inc., La Jolla, CA, USA), using Kruskal-Wallis test with Dunn’s post hoc test to compare different columns. A p-value smaller than 0.05 was regarded as statistically significant.

## Results

### SPRY4 immunostaining in antrum of Kit^K641E/K641E^ and Kit^WT/K641E^ mice

**Fig 1:**
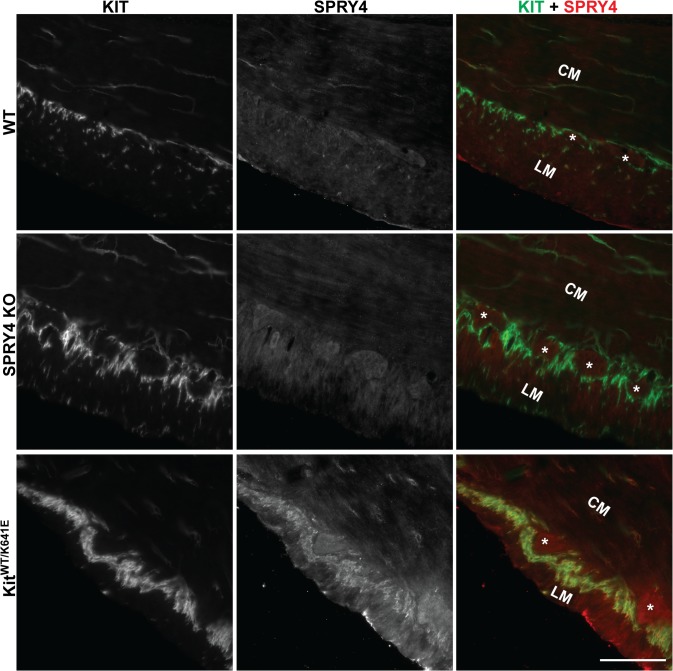
SPRY4-ir in the ICC of 3-month-old *Kit* ^*WT/K641E*^ antrum. Widefield microscopy, sequential channels acquisitions. Left column: KIT immunoreactivity (-ir) ICC in WT, Spry4 KO and KitWT/K641E. Middle column: SPRY4-ir in the 3 genotypes. Right column: merged images: KIT-ir and SPRY4-ir displayed in green and in red, respectively. SPRY4-ir was detected in the KIT-ir ICC solely in KitWT/K641E mice. Abbreviations: LM: longitudinal muscle layer, CM: circular muscle layer, *: location of myenteric plexus, scale bar: 100μm.

This study was based on our previous observation of elevated *Spry4* expression in *Kit* ^*K641E/K641E*^ antrum as compared to WT mice [19]. Here, we confirmed, in 10-day-old animals (P10), strong *Spry4* expression in *Kit* ^*K641E/K641E*^ antrum compared to WT littermates, while no *Spry4* expression could be detected in *Spry4* KO or *Kit* ^*K641E/K641E*^ *-Spry4* KO antrum (S1A Fig). In 3-month-old animals, SPRY4-ir was detected in the ICC of *Kit* ^*WT/K641E*^ animals, but not in WT nor in *Spry4* KO animals (Fig 1). These data are in line with our previous observations [19]. SPRY4 immunoreactivity (-ir) was also detected in the ICC of P10 *Kit* ^*K641E/K641E*^ mice, in contrast to WT and *Spry4* KO mice (S1B Fig).

To validate the SPRY4 antibody used in this study (Table 3), preabsorption with an immunogenic peptide was performed on P10 *Kit* ^*K641E/K641E*^ antrum to assess the disappearance of SPRY4-ir signal after preabsorption (S2 Fig). Furthermore, SPRY4-ir was undetectable in *Kit* ^*K641E/K641E*^ *-Spry4 KO* mice (S1B Fig), establishing it’s specific and suitability for IF.

### Confirmation of dwarfism and polysyndactyly in our Spry4 KO model

*Spry*4 KO mice had a significantly lower weight compared to WT littermates at both P10 and 3 month old (S3A and S3B Fig). Furthermore, some *Spry4* KO animals presented with polysyndactily (S3C Fig).

### Significant increase of ICC area in antrum of 3-month-old Spry4 KO mice

Phosphodiesterase 3A (PDE3A) has previously been identified as an ICC marker [19,32]. Double immunofluorescence staining using KIT (rabbit) and PDE3A (sheep) antibodies (Table 3) confirmed the concordance of these markers in 3-month-old antrum (S4 Fig). PDE3A-ir filled in the cytoplasm while KIT-ir was to a large extend present at the cell membrane but the two clearly labelled the very same cells (S4 Fig). Since many antibodies used in this study (Table 3) have been raised in rabbit, the sheep PDE3A antibody was preferred as ICC marker for subsequent double immunofluorescence studies.

**Fig 2:**
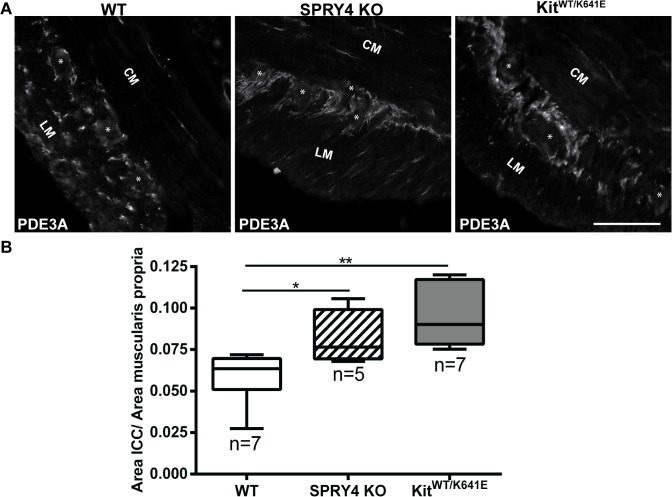
ICC hyperplasia in the antrum of 3-month-old *Spry4* KO and *Kit* ^*WT/K641E*^. A) Widefield microscopy acquisitions. PDE3A immunoreactivity (-ir) highlights ICC in the antrum of 3-month-old WT, *Spry4* KO and *Kit* ^***WT/K641E***^ mice. B) Ratio of PDE3A-ir ICC area in the antrum muscularis propria. Abbreviations: LM: longitudinal muscle layer, CM: circular muscle layer, *: location of myenteric plexus, scale bar: 100μm. P-values (Kruskal-Wallis with Dunn’s post hoc) *: p<0.05, **: p<0.01

Quantification of the PDE3A-ir ICC density in the musculature was performed on the entire circumference of the antrum, by normalizing the area of PDE3A-ir positive signal to the total muscularis propria area. Compared to WT littermates, 3-month-old *Spry4* KO animals showed a significant increase in PDE3A-ir ICC area, similar to *Kit* ^*WT/K641E*^ mice (Fig 2) while in P10 *Spry4* KO animals, no difference was observed in the PDE3A-ir ICC area compared to their WT littermates (S5 Fig). In contrast, P10 homozygous *Kit* ^*K641E/K641E*^ already exhibited a massive PDE3A-ir ICC hyperplasia (S5 Fig), replacing the entire longitudinal muscle layer in the antrum. As these homozygous mice died before weaning, heterozygous *Kit* ^*WT/K641E*^ mice, which display a milder phenotype of ICC hyperplasia, were used for subsequent comparative studies in adult animals.

### No change detected in pERK in antrum ICC of Spry4 KO mice at P10 and 3 months

**Fig 3:**
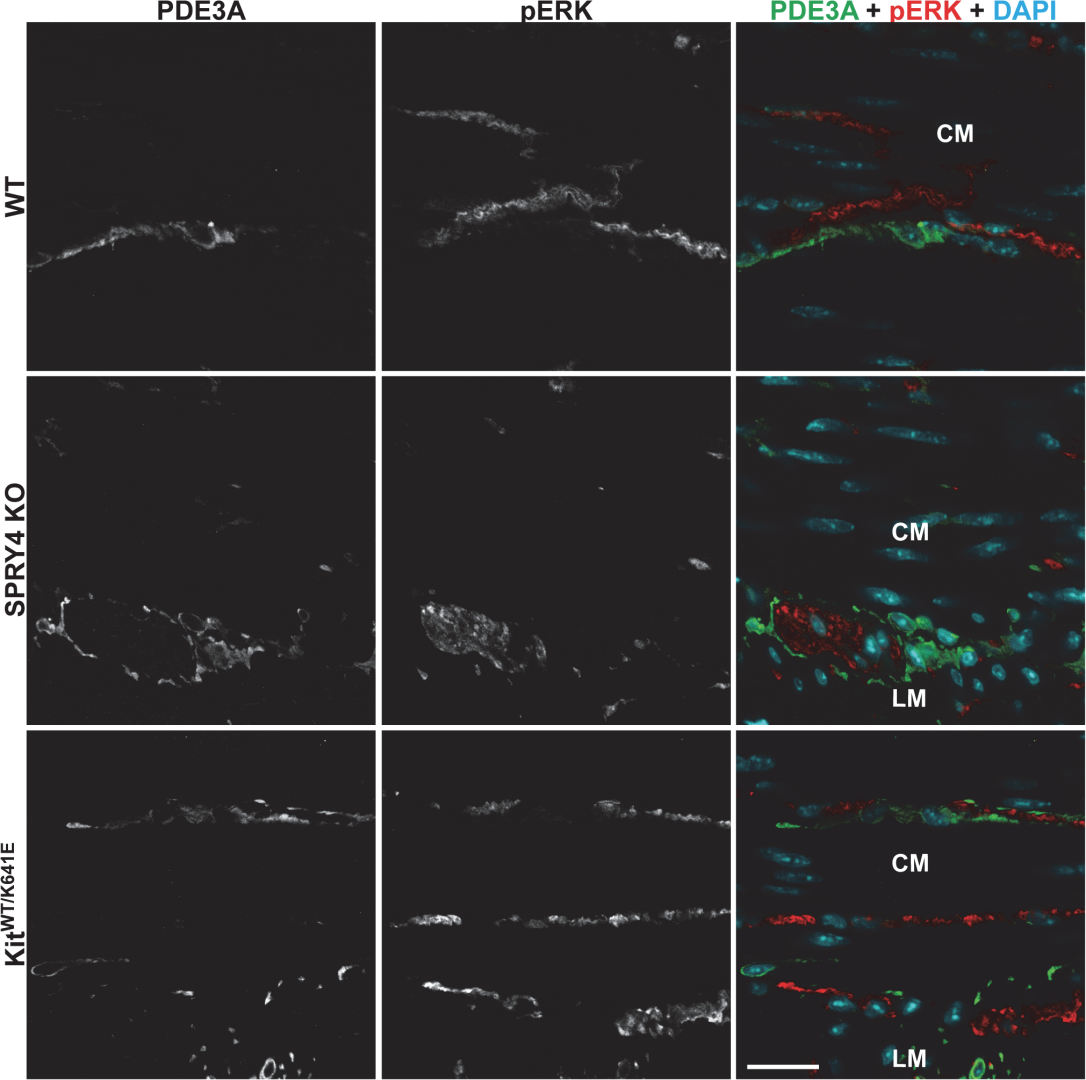
pERK-ir in nerve fibers but undetectable in ICC of 3-month-old WT, *Spry4* KO and *Kit* ^*WT/K641E*^ antrum. Confocal microscopy, sequential channels acquisitions. *Left column*: grey scale images of PDE3A immunoreactivity (-ir) ICC. *Middle column*: grey scale images of pERK-ir. *Right column*: merged images. PDE3A-ir and pERK-ir are displayed in green and in red, respectively, with nuclear counterstain (DAPI) in blue. pERK-ir (red) was consistently detected in myenteric plexus and in nerve fibers in the muscularis propria but not in PDE3A-ir ICC (green). Abbreviations: LM: longitudinal muscle layer, CM: circular muscle layer, scale bar: 20μm

With Sprouty proteins being known as negative regulators of the ERK pathway [14], we hypothesized that phosphorylation of ERK might be elevated in the hyperplastic ICC of the *Spry4* KO animals compared to WT mice. We observed pERK-ir in PDE3A-ir ICC of P10 *Kit* ^*K641E/K641E*^ animals, but not in WT nor in *Spry4* KO littermates (S6 Fig). In 3-month-old animals, pERK-ir was not detected in PDE3A-ir ICC of *Spry4* KO or *Kit* ^*WT/K641E*^ antrum. Conversely, in all genotypes at any age, robust pERK-ir was consistently detected in the myenteric plexus and in intramuscular nerve fibers adjacent to PDE3A-ir ICC in the same field of view (Fig 3) and was thus regarded as an internal positive control.

### No compensation by other SPRYS for the loss of SPRY4 in antrum

**Fig 4:**
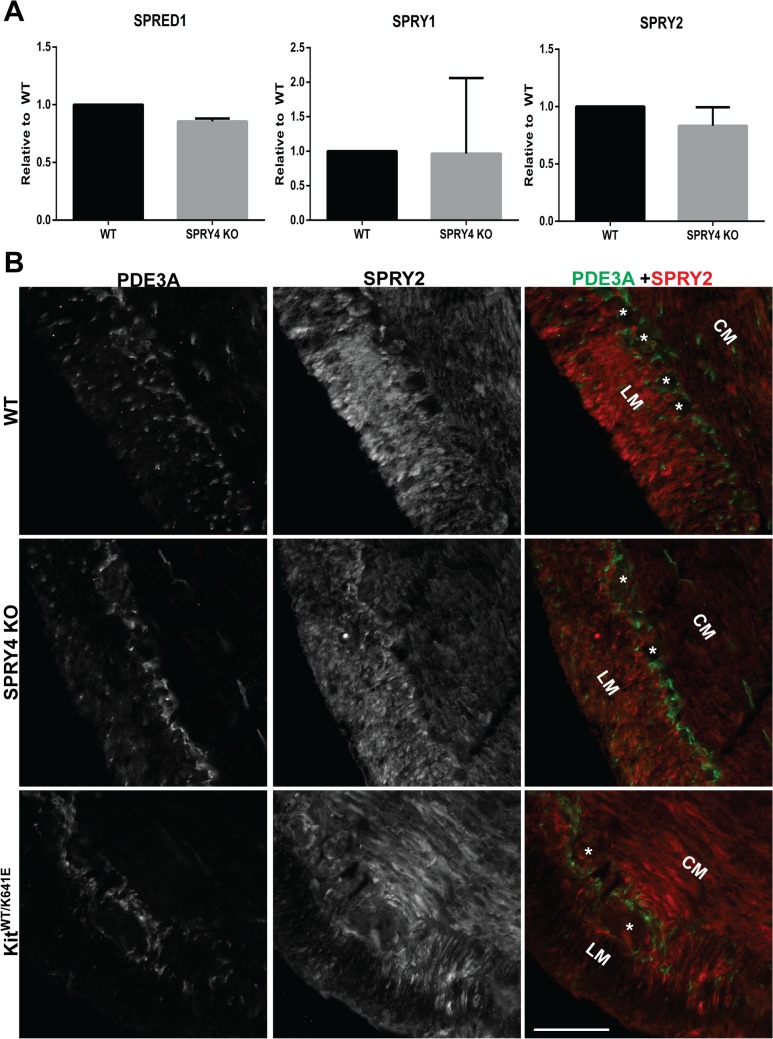
No compensation mechanism detected in *Spry4* KO animals. A) qPCR of WT and *Spry4* KO P10 antrum for *Spred1*, *Spry1* and *Spry2*. B) Widefield microscopy, sequential channels acquisitions. Immunofluorescence of 3-month-old antrum. *Left column*: PDE3A-ir ICC in WT, *Spry4* KO and *Kit* ^***WT/K641E***^. *Middle column*: SPRY2-ir in in the 3 genotypes. *Right column*: merged images: PDE3A-ir and SPRY2-ir displayed in green and in red, respectively. SPRY2-ir was consistently detected in smooth muscle cells of the muscularis propria but not in PDE3A-ir ICC. Abbreviations: LM: longitudinal muscle layer, CM: circular muscle layer, *: location of myenteric plexus, scale bar: 100μm.

We next wondered if compensation mechanisms for the loss of SPRY4 would be present. Therefore, we tested by qPCR the expression levels of SPRY1, SPRY2 and SPRED1 but none of these genes showed any upregulation in *Spry4* KO antrum (Fig 4A). Additionally, SPRY2-ir was detected only in the smooth muscle cells of antrum and not adjacent PDE3A-ir ICC (Fig 4B, S7 Fig).

### No change in other signaling pathways in ICC of 3-month-old antrum

**Fig 5:**
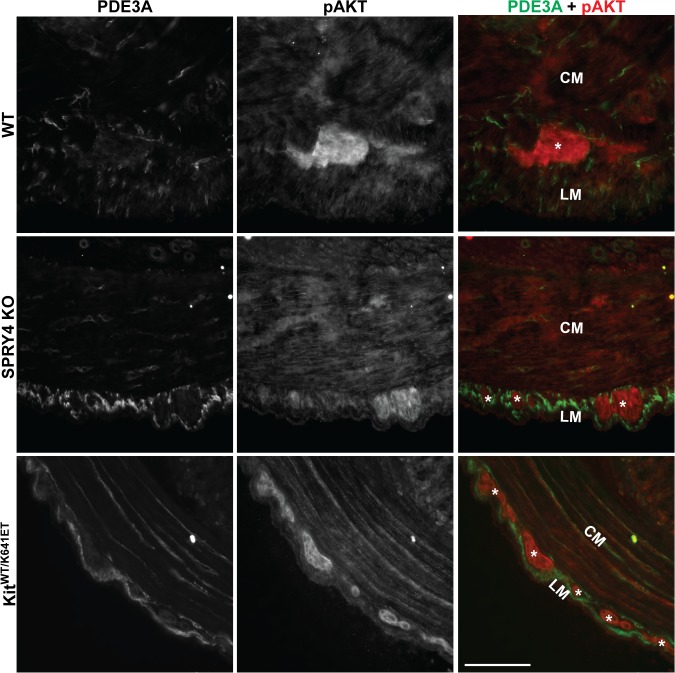
No detectable pAKT-ir in ICC of WT, *Spry4* KO and *Kit* ^*WT/K641E*^ antrum. Widefield microscopy, sequential channels acquisitions. *Left column*: PDE3A immunoreactivity (-ir) ICC in WT, *Spry4* KO and *Kit* ^***WT/K641E***^. *Middle column*: pAKT-ir in the 3 genotypes. *Right column*: merged images: PDE3A-ir and pAKT displayed in green and in red, respectively. pAKT (red) was consistently detected in myenteric plexus and nerve fibers in the muscularis propria but not in PDE3A-ir ICC (green). Abbreviations: LM: longitudinal muscle layer, CM: circular muscle layer, *: myenteric plexus, scale bar: 100μm.

A role of SPRY4 in PI3K/AKT/mTOR signaling has been established in other models [33,34]. pAKT-ir has previously been reported in the hyperplastic ICC layer in P10 *Kit* ^*K641E/K641E*^ antrum [35] and (S8 Fig). In 3 month old animals of all genotypes, pAKT-ir was detected only in the myenteric plexus but not in PDE3A-ir ICC (Fig 5).

**Fig 6:**
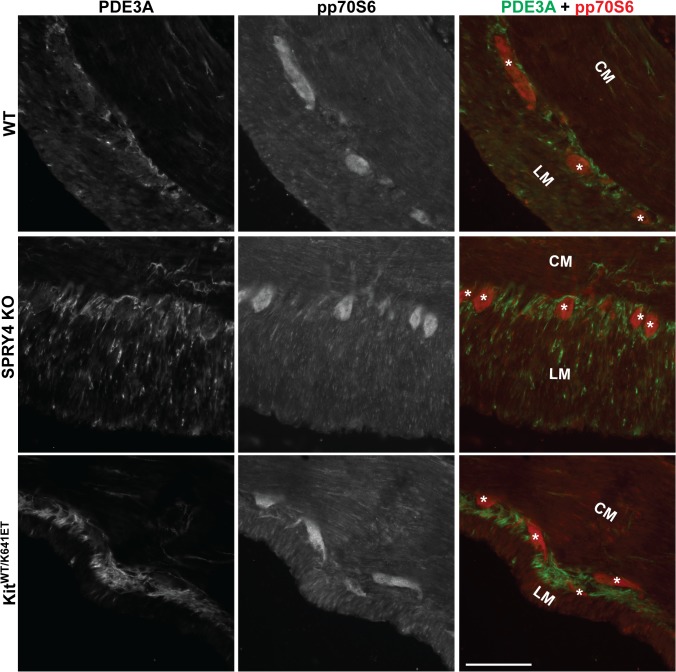
No detectable pp70S6 in ICC of antrum of WT, *Spry4* KO or *Kit* ^*WT/K641E*^. Widefield microscopy, sequential channels acquisitions. *Left column*: PDE3A immunoreactivity (-ir) ICC in WT, *Spry4* KO and *Kit* ^***WT/K641E***^ antrum. *Middle column*: pp70S6-ir for each genotype. *Right column*: merged images: PDE3A and pp70S6-ir displayed in green and red, respectively. pp70S6 was consistently detected in myenteric plexus and nerve fibers in the muscularis propria but not in PDE3A-ir ICC. Abbreviations: LM: longitudinal muscle layer, CM: circular muscle layer, *: location of myenteric plexus, scale bar: 100μm

To further investigate a possible involvement of mTOR pathway, we performed IF for the active form of p70S6, a protein downstream of mTOR, phospho-p70S6 (pp70S6). pp70S6-ir. Was detected in the hyperplastic PDE3A-ir ICC layer of P10 *Kit* ^*K641E/K641E*^ animals (S9 Fig) but, in all other genotypes, at both P10 and 3 month old, pp70S6-ir was detected only in the myenteric plexus of the antrum but not in PDE3A-ir ICC (Fig 6).

**Fig 7:**
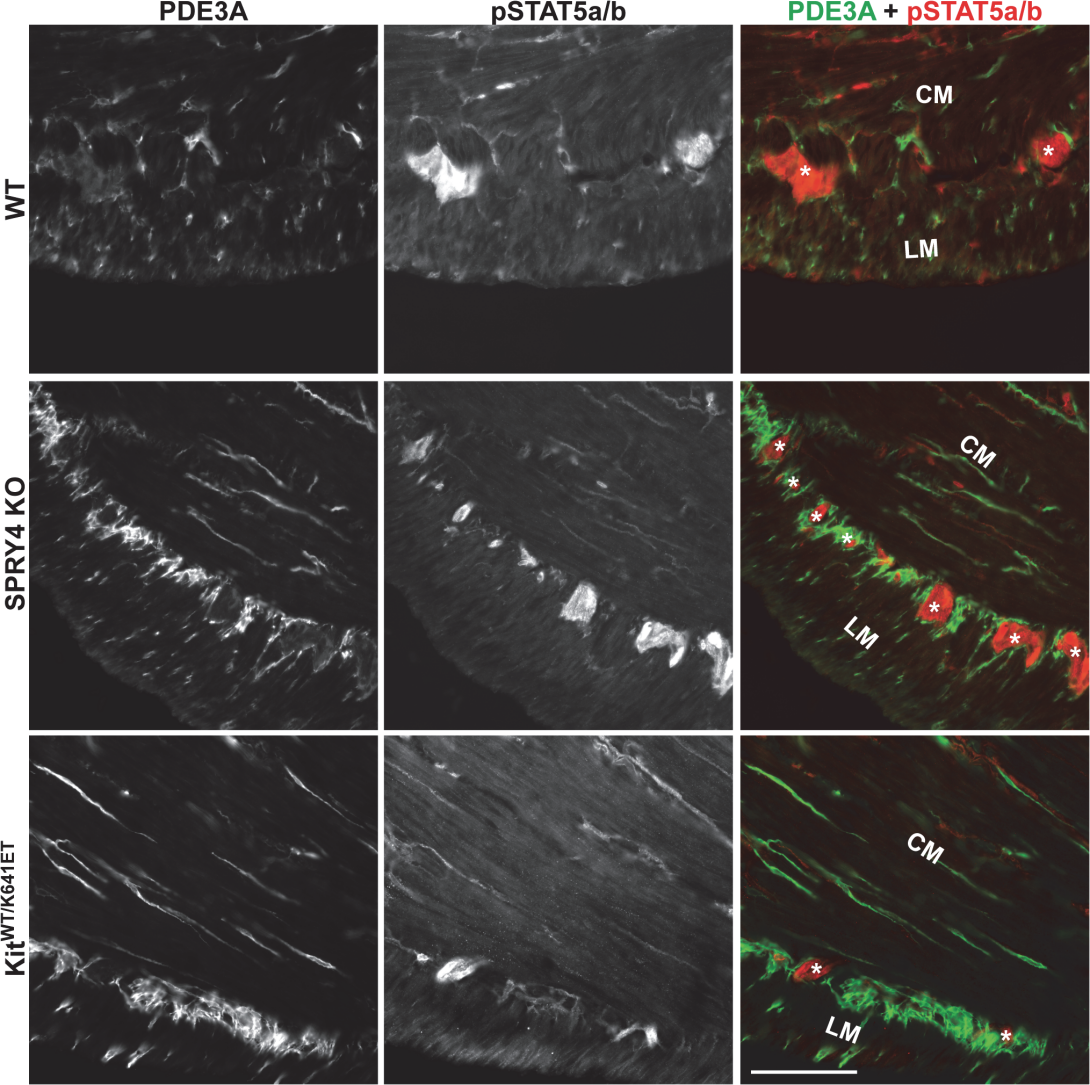
No detectable pSTAT5a/b in ICC of antrum of WT, *Spry4* KO or *Kit* ^*WT/K641E*^. Widefield microscopy, sequential channels acquisitions. *Left column*: PDE3A immunoreactivity (-ir) ICC in WT, *Spry4* KO and *Kit* ^***WT/K641E***^ antrum. *Middle column*: pSTAT5a/b-ir for each genotype. *Right column*: merged images: PDE3A-ir and pSTAT5a/b-ir displayed in green and red, respectively. pSTAT5a/b was consistently detected in myenteric plexus and nerve fibers in the muscularis propria but not in PDE3A-ir ICC. Abbreviations: LM: longitudinal muscle layer, CM: circular muscle layer, *: location of myenteric plexus, scale bar: 100μm

Although SPRY4 has not been directly implicated in the JAK/STAT pathway, this pathway is also involved upon KIT receptor activation [35–38]. Immunoreactivity for the active (phosphorylated) form of STAT5 (pSTAT5-ir) was detected in the hyperplastic PDE3A-ir ICC layer of P10 Kit^K641E/K641E^ antrum (S10 Fig). Conversely, pSTAT5a/b-ir was not detected in the PDE3A-ir ICC of P10 and 3 month old antrum of WT, *Spry4* KO or *Kit* ^*WT/K641E*^ (Figs 7, S10).

### Hypoganglionosis in antrum of both Spry4 KO and Kit^WT/K641E^ mice

**Fig 8:**
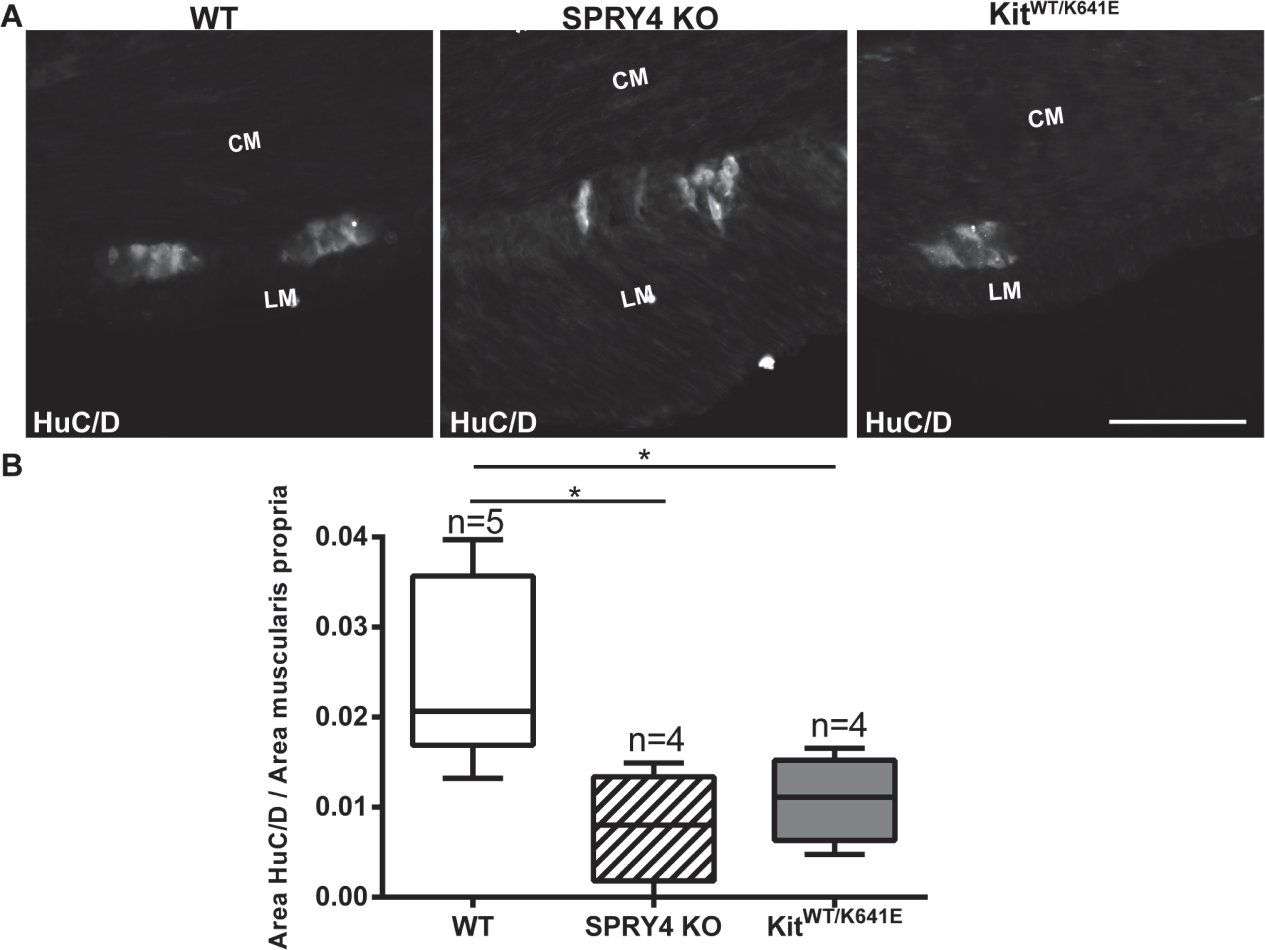
Decrease in myenteric neurons area in antrum of *Spry4* KO and *Kit* ^*WT/K641E*^ mice. A) Widefield microscopy acquisitions. HuC/D-ir highlights soma of myenteric neurons in antrum of 3-month-old WT, *Spry4* KO and *Kit* ^***WT/K641E***^ mice. B) Ratio of HuC/D-ir area in antrum muscularis propria. Abbreviations: LM: longitudinal muscle layer, CM: circular muscle layer, scale bar: 100μm. P-values (Kruskal-Wallis with Dunn’s post hoc), *: p<0.05

Hyperganglionosis has been reported in the colon of *Spry2* KO mice [39]. We thus wondered whether the enteric nervous system (ENS) could similarly be affected in *Spry4* KO mice. HuC/D-ir was used for the detection of myenteric neural bodies [40] in the antrum of 3-month-old mice (Fig 8A). HuC/D-ir positive area was normalized for the muscularis propria area. Both *Spry4* KO and *Kit* ^*WT/K641E*^ mice showed a decrease of neural bodies in antrum, compared to WT (Fig 8B).

### No change in ICC or signaling pathways in small intestine of 3-month-old Spry4 KO mice

**Fig 9:**
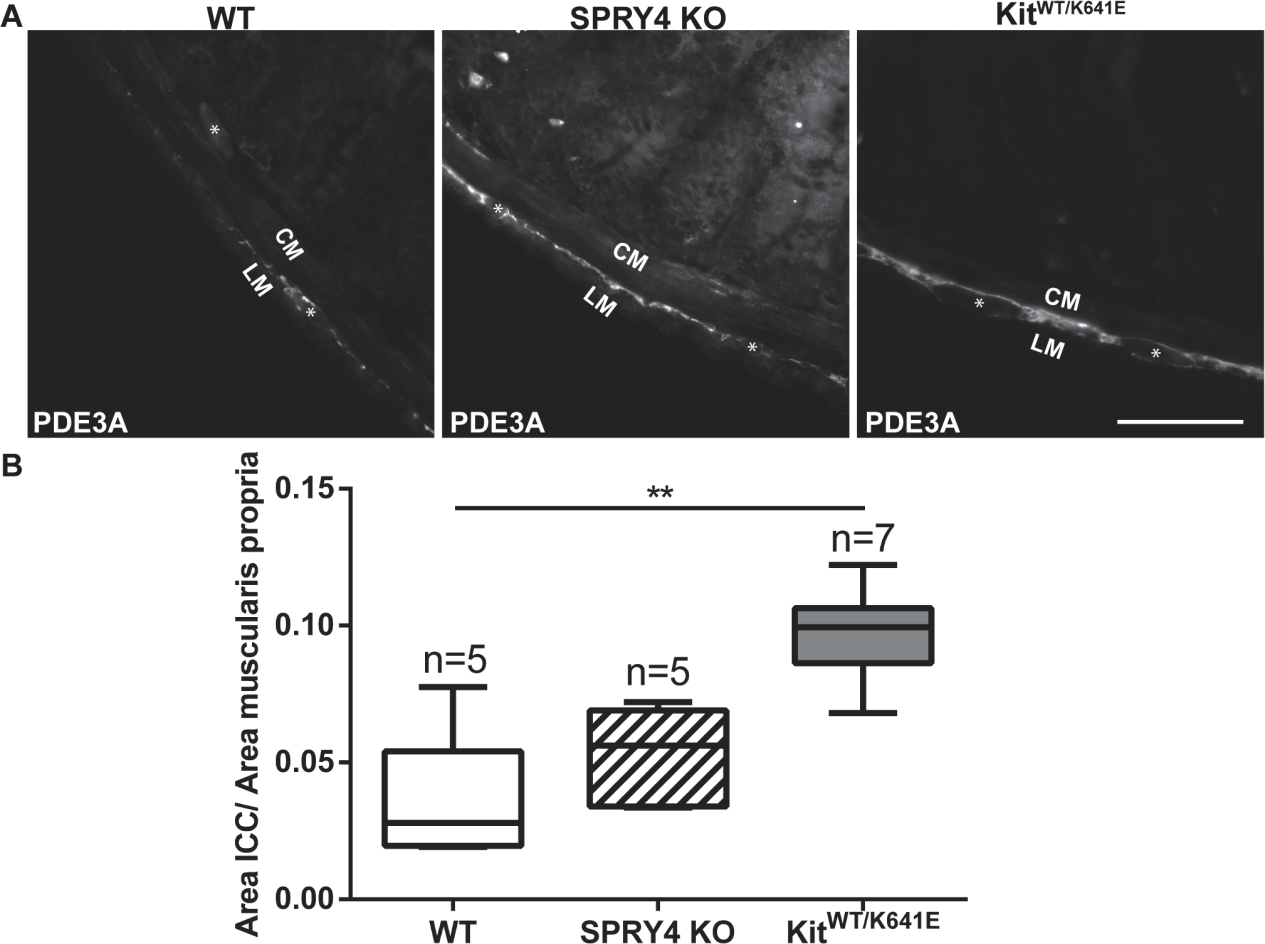
ICC hyperplasia in small intestine of *Kit* ^*WT/K641E*^. A) Widefield microscopy acquisitions. PDE3A immunoreactivity (-ir) highlights ICC in the small intestine of 3-month-old WT, *Spry4* KO and *Kit* ^***WT/K641E***^ mice. B) Ratio of PDE3A-ir ICC area in muscularis propria of small intestine. Abbreviations: LM: longitudinal muscle layer, CM: circular muscle layer, *: location of myenteric plexus, scale bar: 100μm. P-values (Kruskal-Wallis with Dunn’s post hoc) **: p<0.01

Hyperplasia of ICC in *Kit* ^*K641E*^ animals is not restricted to the antrum [22]. Hence, we also performed PDE3A immunostaining in order to quantify ICC in the small intestine (Fig 9, as described above). A trend towards an increase of PDE3A-ir ICC area in small intestine of *Spry4* KO mice did not reach statistical significance (n = 5–7 animals per group) while, the PDE3A-ir ICC area was significantly increased in *Kit* ^*WT/K641E*^ small intestine (Fig 9).

In the small intestine, in all genotypes, pERK-ir was detected only in the myenteric plexus (S11 Fig), and SPRY2 solely in the smooth muscle layers (S12 Fig). Quantification of neuronal bodies using HuC/D IF in 3-month-old small intestine did not show significant difference for any genotype (S13 Fig).

### Significant increase of ICC area in colon of 3-month-old Spry4 KO mice

**Fig 10:**
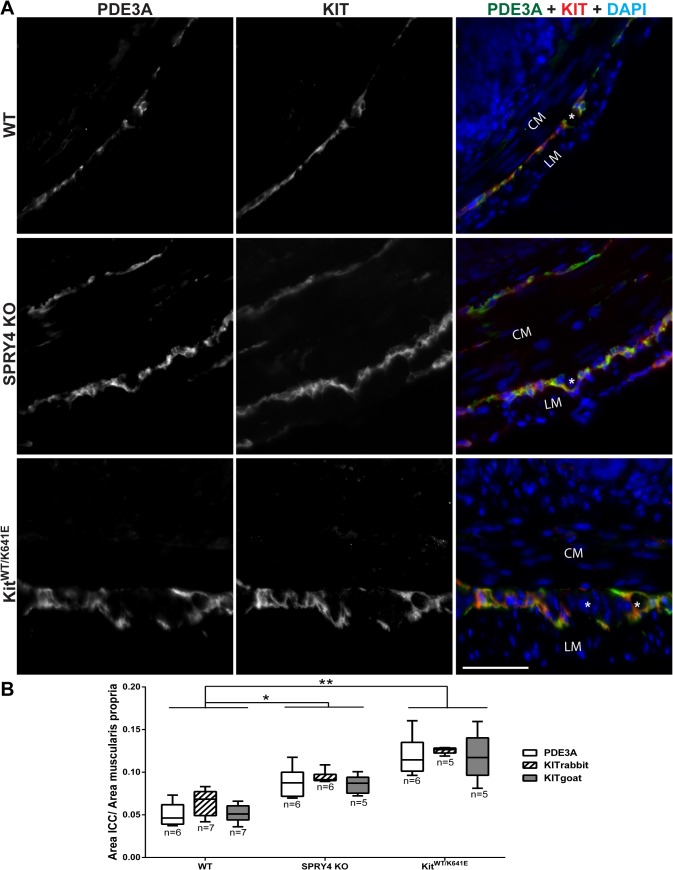
ICC hyperplasia in colon of 3-month-old *Spry4* KO and *Kit* ^*WT/K641E*^. A) Widefield microscopy acquisitions. PDE3A immunoreactivity (-ir) and KIT-ir (rabbit) highlight ICC in the colon of 3-month-old WT, *Spry4* KO and *Kit* ^***WT/K641E***^ mice. B) Quantification of PDE3A-ir, KIT-ir (rabbit) and KIT-ir (goat) ICC area in the muscularis propria of 3-month-old WT, *Spry4 KO* and *Kit* ^***WT/K641E***^ colon (n = 5–7 animals per group). Single IF staining performed on adjacent sections. The 3 antibodies identified similarly a significant increase in ICC area in *Spry4* KO (p value < 0.05 for each staining) and in *Kit* ^***WT/K641E***^ colon (PDE3A a KIT goat p < 0.01, KIT rabbit p value < 0.001) compared to WT colon. No significant difference was observed between *Spry4 KO* and *Kit* ^***WT/K641E***^ (p value PDE3A = 0.2509, p value KIT rabbit = 0.2668, p value KIT goat = 0.3572). Within each genotype, differences between the 3 antibodies were not significant. Abbreviations: LM: longitudinal muscle layer, CM: circular muscle layer, *: location of myenteric plexus, scale bar: 50μm. P-values (Kruskal-Wallis with Dunn’s post hoc) *: p<0.05, **: p<0.01

Double immunofluorescence staining using KIT (rabbit) and PDE3A (sheep) antibodies (Table 3) indicated an increased ICC area in both *Spry4* KO and *Kit* ^*WT/K641E*^ colon (Fig 10A). Quantitative assessment of ICC area was performed by single IF staining using KIT-ir (two different, rabbit and goat, KIT antibodies—Table 3) and PDE3A-ir on adjacent sections of colon for WT, *Spry4* KO and *Kit* ^*WT/K641E*^ genotypes (n = 5–7 animals per group). Within each genotype, the 3 antibodies gave concordant results, with non-significant differences between antibodies. The 3 antibodies identified similarly a significant increase in ICC area in *Spry4* KO colon (p value <0.05 for each antibody) and in *Kit* ^*WT/K641E*^ colon (KIT rabbit p value <0.001, KIT goat and PDE3A p value < 0.01) compared to WT. Although ICC hyperplasia appeared more pronounced in *Kit* ^*WT/K641E*^ than in *Spry4* KO, differences between *Kit* ^*WT/K641E*^ and *Spry4* KO were not significant (p value PDE3A = 0.2509, p value KIT rabbit = 0.2668, p value KIT goat = 0.3572).

Similarly to antrum and small intestine, pERK-ir was only detected in the myenteric plexus (S14 Fig), and SPRY2-ir solely in the smooth muscle layers (S15 Fig). HuC/D-ir quantification in the colon did not show any difference between genotypes (S16 Fig).

### Significant increase in total transit time in Spry4 KO and Kit^WT/K641E^mice

**Fig 11:**
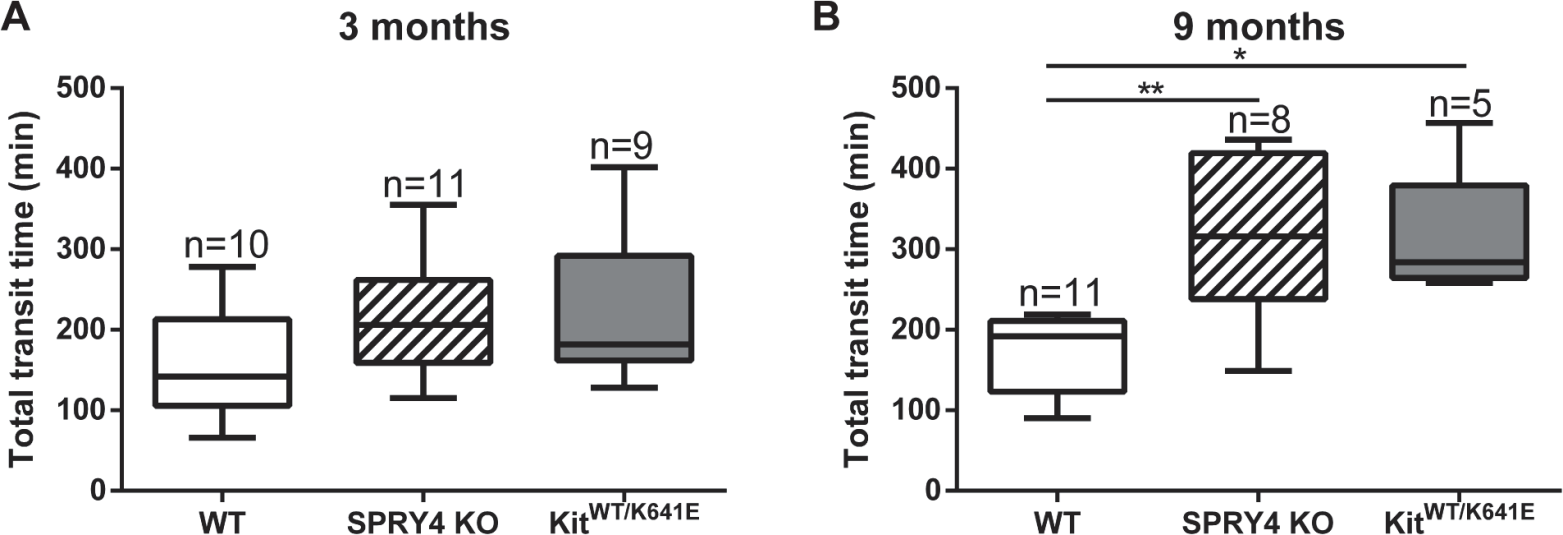
Total digestive transit time is delayed in 9 month old *Spry4* KO and *Kit* ^*WT/K641E*^ mice compared to WT animals. Total transit time was determined using the Carmine red method. The delayed in transit time was not significant in 3-month-old *Spry4* and *Kit* ^*WT/K641E*^ animals (A) but delay became significant by 9 months of age (B). P-values (Kruskal-Wallis test with Dunn’s post-hoc) *: p<0.05, **: p<0.01.

ICC regulate the peristaltic movement of the gastrointestinal tract [41]. We wondered if ICC hyperplasia in *Spry4* KO and *Kit* ^*WT/K641E*^ mice, could impact the gut propulsive function. We thus tested the transit time using the carmine red method in 3 and 9 month old animals. At 3 month of age, total transit time was similar between genotypes (Fig 11A). Conversely, at 9 months, total transit time was significantly increased in both *Spry4* KO and *Kit* ^*WT/K641E*^, compared to their WT littermates (Fig 11B). Changes in total transit time were not related to the length of the small intestine since it did not differ between genotypes (S17 Fig).

## Discussion

Here, we report a hyperplasia of ICC in both antrum and colon of SPRY4 KO mice. ICC were labeled using KIT-ir, the reference ICC marker [42] & (Fig 1 and Fig 10, S1 Fig and S4 Fig), but also using PDE3A-ir. *Pde3a* belongs to the gene expression profile of KIT-ir ICC cell-sorted in the mouse small intestine [32]. *Pde3a* also appeared among the genes upregulated in *Kit* ^*K641E/K641E*^ antrum, presenting massive hyperplasia of KIT-ir ICC, compared to WT littermate and PDE3A-ir localized in KIT-ir ICC in the antrum of WT and Kit^K641E^ (homozygous and heterozygous) mice. PDE3A-ir was therefore regarded as a novel marker for the KIT-ir ICC in the mouse gut [19]. Although PDE3A-ir has not gained yet a wider acceptance as ICC marker, to the best of our knowledge, our original claim [19] remains so far unchallenged in the literature. In the present study, we used high resolution confocal imaging to show, at the level of individually identified cells, that PDE3A-ir decorates electively the KIT-ir ICC, in *Spry4* KO antrum, as well as in WT and *Kit* ^*WT/K641E*^ antrum (S4 Fig). We also quantitated the ICC area using KIT-ir (2 different antibodies) and PDE3A-ir in *SPRY4* KO colon, as well as in WT and *Kit* ^*WT/K641E*^ (Fig 10). Within each genotype, KIT-ir and PDE3A-ir values proved to be concordant and, both PDE3A-ir and KIT-ir similarly detected the well-established ICC hyperplasia in *Kit* ^*WT/K641E*^ colon and revealed a significant ICC hyperplasia in *Spry4* KO colon compared to WT. Although rare events, i.e. the possible occurrence of tiny populations of KIT negative PDE3A-ir cells or KIT-ir PDE3A negative cells, cannot be totally ruled out by these experiments, the presence of PDE3A-ir selectively in the KIT-ir ICC in the 3 genotypes studied and the ability of PDE3A-ir to detect, to the same extend as KIT-ir, the predicted ICC hyperplasia in the reference model for KIT-ir ICC hyperplasia, *Kit* ^*K641E*^ mice, and in Spry4 KO colon comfort our view [19] that PDE3A-ir represents globally a valuable marker for the Kit-ir ICC in the WT mouse gut and in ICC hyperplasia models.

Sprouty proteins are known negative regulators of the ERK pathway [14], a pathway involved in cell division, survival and transformation [43], we anticipated that ERK would play a critical role in the development of ICC hyperplasia. ICC represent less than 1% of the total cell population of the highly heterogeneous gut wall. Modifications in gene or protein expression occurring in ICC might thus vanish in the ambient ‘noise’ when using techniques which require tissue homogenization, e.g. qPCR or Western blot. Therefore, immunoreactivity was used to study signaling pathways in situ with resolution of the different cell types. Surprisingly, pERK was undetectable in the ICC of *Spry4* KO mice. In order to exclude compensatory upregulation of other Sprouty family members, we performed qPCR of the antrum for *Spry1*, *Spry2*, and *Spred1*. None of these genes were upregulated in *Spry4* KO antrum. No commercially available antibodies for SPRY1 and SPRED1 were fond suitable for IF on our material while SPRY2-ir showed no change between the different genotypes. Nevertheless, despite these limitations, our data suggest that no compensation by other SPRYs appears to occur. Recently, several papers indicated that SPRY4 can play a role in other signaling pathways [33,34,44,45]. Hence, pAKT and pp70S6 IF were performed in order to investigate the PI3K/AKT/mTOR and pSTAT5a/b for the JAK/STAT pathways, but no detectable differences in immunoreactivity could be seen between the different genotypes.

Interestingly, the phenotype of *Spry4* KO mice is reminiscent of the phenotype of heterozygous *Kit* ^*WT/K641E*^ mice (Table 4). Both show hyperplasia of the ICC in antrum with no detectable changes in signaling pathways tested in this study. This contrasts with the homozygous *Kit* ^*K641E/K641E*^ phenotype, which shows complete replacement of the longitudinal layer of the antrum by a hyperplastic ICC layer in which pERK-ir, pAKT-ir, pp70S6-ir and pSTAT5a/b-ir is detectable. Rubin et al demonstrated elevated tyrosine phosphorylation of the KIT receptor in *Kit* ^*K641E/K641E*^ mice [22]. Hence, strong KIT phosphorylation in homozygous *Kit* ^*K641E/K641E*^ mice may lead to a strong activation of the signaling pathways, while heterozygous *Kit* ^*WT/K641E*^ mice would have lower levels of KIT phosphorylation and therefore lesser activation of the downstream signaling pathways than *Kit* ^*K641E/K641E*^ animals. In GIST882 cells, a human GIST cell line carrying the same K-to-E substitution as the *Kit* ^*K641E*^ mice [10], the link between ERK phosphorylation and upregulation of SPRY4 has been established [25,46]. One can only speculate that a small increase in ERK phosphorylation in the ICC of heterozygous *Kit* ^*WT/K641E*^ mice might well fall below the detection threshold of immunohistochemistry which readily picks up the strong ERK phosphorylation in ICC in *Kit* ^*K641E/K641E*^ mice and in the ENS in all genotypes.

**Table 4:** Summary of results in 3-month-old SPRY4 KO and Kit^*wr/K641E*^ animals compared to controls.

Genotype	*Spry4* KO	*Kit* ^*WT/K641E*^
***ICC antrum***	Increased	Increased
***ICC colon***	Increased	Highly increased
***ICC small intestine***	Comparable	Increased
***HuC/D antrum***	Decreased	Decreased
***Total transit time***	Increased	Increased

Despite the fact that SPRY4-ir was not detectable in ICC in the postnatal WT gut [19], Fig 1 & S1 Fig, this study revealed ICC hyperplasia in *Spry4* KO mice, raising the possibility that *Spry4* might play a role during ICC embryonic development. Noteworthy, *Spry4* expression, detected by in situ hybridization, has been reported in mouse embryo, in stomach at embryonic day 11.5 (E11.5) and in intestine at E12.5 and E14.5 [47], i.e. around the time where ICC differentiation starts [48–50].

In a similar perspective, Taketomi et al reported that SPRY2 deficient mice exhibit hyperganglionosis in the colon, although the exact mechanism remains unclear [39]. Since, SPRY2 and SPRY4 belong to the same protein family [12,14], the ENS in *Spry4* KO mice was also investigated. Surprisingly, the antrum of *Spry4* KO mice presented a hypoganglionosis, while other parts of the gastrointestinal tract appeared normoganglionic. The same feature was also seen in *Kit* ^*WT/K641E*^ antrum, adding to the similarities between *Spry4* KO and *Kit* ^*WT/K641E*^ (Table 4). Migration of the neural crest cells forming the ENS is completed by E15 in mice [51]. Hence, the ENS alterations observed in *Spry4* null and *Kit* ^*WT/K64E*^ animals after birth must originate during embryonic gut development.

The present study focused on the postnatal gut phenotype of *Spry4* KO mice and further studies are clearly needed to unravel the time windows at which *Spry4* is expressed in the different cell types and the underlying signaling pathways in the developing gut.

ICC are the pacemaker cells of the gastrointestinal tract, coordinating the contractility of the gastrointestinal muscle layers [41,52]. Bellier et al reported that PRM/Alf mice, which exhibit a higher number of ICC and increased intestine length, have a total gastrointestinal transit time similar to their WT littermates, implying a faster transit [53]. In contrast to PRM/Alf mice, the length of intestine in *Spry4* deficient and *Kit* ^*WT/K641E*^ mice was similar to WT littermates and the increase in ICC was associated with a significantly delayed total gastrointestinal transit in aging (9 month old)—but not in younger (3-month-old) animals. This provides an original clue that, besides roles during development, *Spry4* and *Kit* ^*K641E*^ may play additional roles during the aging process. Digestive transit is a very complex, multifactorial, process, and further studies (e.g. electrophysiology, microbiome, etc.) will be required to unravel the precise mechanism underlying these observations. Noteworthy, constipation, albeit a fairly common and unspecific complain, is a frequent symptom in human adult GIST patients [54–57].

In summary, we have shown that *SPRY4* loss of function was associated with ICC hyperplasia in antrum and colon. The *Spry4* KO mice bear striking similarities with the *Kit* ^*WT/K641E*^ oncogenic mice, and in both models, ICC hyperplasia was associated with a delayed total gastrointestinal transit time in aging mice.

## Supporting Information

S1 Fig *Spry4* mRNA expression and immunoreactivity in P10 antrum. A) qPCR analysis of *Spry4* mRNA levels in the postnatal (P10) mouse antrum showing a significant increase in *Spry4* expression in *Kit* ^*K641E/K641E*^ antrum compared to WT, while *Spry4* expression was undetectable in *Spry4* KO or *Kit* ^*K641E/K641E*^ *-Spry4* KO. P-values (Kruskal-Wallis test with Dunn’s post-hoc) **: p<0.01, ***: p<0.001. B) Immunofluorescence for KIT immunoreactivity (-ir) and SPRY4-ir in P10 antrum. Widefield microscopy, sequential channels acquisitions. *Left column*: grey scale images of KIT-ir ICC. *Middle column*: grey scale images of SPRY4-ir. *Right column*: merged images. KIT-ir and SPRY4-ir are displayed in green and in red, respectively. SPRY4-ir (red) was detected in the KIT-ir ICC (green) only in *Kit* ^*K641E/K641E*^ mice but not in WT, *Spry4 KO* or *Kit* ^*K641E/K641E*^ *-Spry4* KO antrum. Abbreviations: LM: longitudinal muscle layer, CM: circular muscle layer, *: myenteric plexus, scale bar: 100μm.
(TIF)

S2 Fig Preabsorption with SPRY4 antigenic peptide abolished SPRY4-ir signal in P10 *Kit* ^*K641E/K641E*^ antrum. A) Control SPRY4-ir (without peptide). SPRY4 antibody preabsorption with B) 1μg or C) 0.1μg immunogenic peptide wiped out the signal. D) Negative control (omission of primary antibody). Scale bar: 50μm.
(TIF)

S3 Fig Dwarfism and polysyndactyly in *Spry4* KO mice. Female and male *Spry4* KO animals showed a significantly lower body weight compared to their WT littermates at A) P10 and B) 3 months of age. C) An example of polysyndactyly in *Spry4* KO animals.
(TIF)

S4 Fig PDE3A-ir in KIT-ir ICC of WT, *Spry4* KO and *Kit* ^*WT/K641E*^ adult mouse antrum. Confocal microscopy, sequential channels acquisitions. *Upper row*: grey scale images of PDE3A immunoreactivity (-ir) ICC. *Second row*: grey scale images of KIT-ir ICC. *Third row*: merged images. PDE3A-ir is displayed in green, KIT-ir in red, with nuclear counterstain (DAPI) in blue. *Bottom row*: Immunofluorescence intensity plots for PDE3A-ir and KIT-ir along the lines drawn across individual cells above, demonstrating that PDE3A-ir and KIT-r were consistently found in the same cells. Abbreviations: LM: longitudinal muscle layer, CM: circular muscle layer, scale bar: 10μm
(TIF)

S5 Fig ICC hyperplasia in antrum of P10 *Kit* ^*K641E/K641E*^ mice. A) Widefield microscopy acquisitions. PDE3A immunoreactivity (-ir) highlights ICC in the antrum of 10 days old WT, *Spry4* KO and *Kit* ^*WT/K641E*^ mice. B) Ratio of PDE3A-ir ICC area in antrum muscularis propria. Abbreviations: LM: longitudinal muscle layer, CM: circular muscle layer, *: location of myenteric plexus, scale bar: 50μm. P-values (Kruskal-Wallis with Dunn’s post hoc), *: p<0.05
(TIF)

S6 Fig pERK present in ICC of P10 *Kit* ^*K641E/K641E*^ antrum. Widefield microscopy, sequential channels acquisitions. *Left column*: PDE3A immunoreactivity (-ir) ICC in WT, *Spry4* KO and *Kit* ^*WT/K641E*^. *Middle column*: pERK-ir in in the 3 genotypes. *Right column*: merged images: PDE3A-ir and pERK-ir displayed in green and in red, respectively. pERK-ir (red) was consistently detected in myenteric plexus and nerve fibers in the muscularis propria of all genotypes. PDE3A-ir ICC (green) which were also pERK-ir were solely detected in *Kit* ^*K641E/K641E*^ mice—and not in the other genotypes. Abbreviations: LM: longitudinal muscle layer, CM: circular muscle layer, *: location of myenteric plexus, scale bar: 50μm.
(TIF)

S7 Fig SPRY2-ir highlights the smooth muscle cells but not the ICC in P10 antrum. Widefield microscopy, sequential channels acquisitions. *Left column*: PDE3A-ir ICC in WT, *Spry4* KO and *Kit* ^*K641E/K641E*^. *Middle column*: SPRY2-ir in in the 3 genotypes. *Right column*: merged images: PDE3A-ir and SPRY2-ir displayed in green and in red, respectively. SPRY2-ir was consistently detected in smooth muscle cells of the muscularis propria but not in PDE3A-ir ICC. Abbreviations: LM: longitudinal muscle layer, CM: circular muscle layer, *: location of myenteric plexus, scale bar: 50μm.
(TIF)

S8 Fig pAKT-ir in ICC of P10 *Kit* ^*K641E/K641E*^ antrum. Widefield microscopy, sequential channels acquisitions. *Left column*: PDE3A immunoreactivity (-ir) ICC in WT, SPRY4 KO and Kit^WT/K641E^. *Middle column*: pAKT-ir in in the 3 genotypes. *Right column*: merged images: PDE3A-ir and pAKT displayed in green and in red, respectively. pAKT (red) was consistently detected in myenteric plexus and nerve fibers in the muscularis propria but solely in PDE3A-ir ICC (green) of *Kit* ^*K641E/K641E*^ animals. Abbreviations: LM: longitudinal muscle layer, CM: circular muscle layer, *: location of myenteric plexus, scale bar: 50μm.
(TIF)

S9 Fig pp70S6-ir in ICC of antrum P10 *Kit* ^*K641E/K641E*^. Widefield microscopy, sequential channels acquisitions.*Left column*: PDE3A immunoreactivity (-ir) ICC in WT, *Spry4* KO and *Kit* ^*K641E/K641E*^ antrum. *Middle column*: pp70S6-ir for each genotype. *Right column*: merged images: PDE3A and pp70S6-ir displayed in green and red, respectively. pp70S6 was consistently detected in myenteric plexus and nerve fibers in the muscularis propria but solely in PDE3A-ir ICC of *Kit* ^*K641E/K641E*^ animals. Abbreviations: LM: longitudinal muscle layer, CM: circular muscle layer, *: myenteric plexus, scale bar: 50μm
(TIF)

S10 Fig pSTAT5a/b-ir in ICC of antrum P10 *Kit* ^*K641E/K641E*^. Widefield microscopy, sequential channels acquisitions. *Left column*: PDE3A immunoreactivity (-ir) ICC in WT, *Spry4* KO and *Kit* ^*K641E/K641E*^ antrum. *Middle column*: pSTAT5a/b-ir for each genotype. *Right column*: merged images: PDE3A and pSTAT5a/b-ir displayed in green and red, respectively. pSTAT5a/b was consistently detected in myenteric plexus and nerve fibers in the muscularis propria but solely in PDE3A-ir ICC of *Kit* ^*K641E/K641E*^ animals. Abbreviations: LM: longitudinal muscle layer, CM: circular muscle layer, *: myenteric plexus, scale bar: 50μm.
(TIF)

S11 Fig No detectable pERK-ir in ICC of WT, *Spry4* KO and *Kit* ^*WT/K641E*^ small intestine of 3-month-old animals. Widefield microscopy, sequential channels acquisitions.*Left column*: PDE3A immunoreactivity (-ir) staining ICC in WT, *Spry4* KO and *Kit* ^*WT/K641E*^. *Middle column*: pERK-ir in the 3 genotypes. *Right column*: merged images: PDE3A-ir and pERK-ir displayed in green and in red, respectively. Abbreviations: LM: longitudinal muscle layer, CM: circular muscle layer, *: location of myenteric plexus, scale bar: 100μm.
(TIF)

S12 Fig SPRY2-ir highlights smooth muscle cells but not ICC in the small intestine of WT, *Spry4* KO or *Kit* ^*WT/K641E*^ adult mice. Widefield microscopy, sequential channels acquisitions.*Left column*: PDE3A-ir ICC in WT, *Spry4* KO and *Kit* ^*WT/K641E*^. *Middle column*: SPRY2-ir in in the 3 genotypes. *Right column*: merged images: PDE3A-ir and SPRY2-ir displayed in green and in red, respectively. SPRY2-ir (red) was consistently detected in the smooth muscle cells of the muscularis propria but not in PDE3A-ir ICC (green). Abbreviations: LM: longitudinal muscle layer, CM: circular muscle layer, *: location of myenteric plexus, scale bar: 100μm.
(TIF)

S13 Fig Myenteric plexus area is similar in the small intestine of *Spry4* KO and *Kit* ^*WT/K641E*^ at 3 months of age. A) Widefield microscopy acquisitions. HuC/D-ir highlights the soma of myenteric neurons in small intestine of 3-month-old WT, *Spry4* KO and *Kit* ^*WT/K641E*^ mice. B) Ratio of HuC/D-ir area in small intestine muscularis propria. Abbreviations: LM: longitudinal muscle layer, CM: circular muscle layer, scale bar: 100μm.
(TIF)

S14 Fig No detectable pERK-ir in ICC of WT, *Spry4* KO and *Kit* ^*WT/K641E*^ colon of 3 month old animals. Widefield microscopy, sequential channels acquisitions.*Left column*: PDE3A immunoreactivity (-ir) staining ICC in WT, *Spry4* KO and *Kit* ^*WT/K641E*^. *Middle column*: pERK-ir in the 3 genotypes. *Right column*: merged images: PDE3A-ir and pERK-ir displayed in green and in red, respectively. Abbreviations: LM: longitudinal muscle layer, CM: circular muscle layer, *: location of myenteric plexus, scale bar: 100μm.
(TIF)

S15 Fig SPRY2-ir highlights smooth muscle cells but not ICC in WT, *Spry4* KO or *Kit* ^*WT/K641E*^ colon at 3 months of age. Widefield microscopy, sequential channels acquisitions.*Left column*: PDE3A-ir ICC in WT, *Spry4* KO and *Kit* ^*WT/K641E*^. *Middle column*: SPRY2-ir in in the 3 genotypes. *Right column*: merged images: PDE3A-ir and SPRY2-ir displayed in green and in red, respectively. SPRY2-ir (red) was consistently detected in the smooth muscle cells of the muscularis propria but not in PDE3A-ir ICC (green). Abbreviations: LM: longitudinal muscle layer, CM: circular muscle layer, *: location of myenteric plexus, scale bar: 100μm.
(TIF)

S16 Fig Myenteric plexus area is unaltered in the colon of 3 month old *Spry4* KO and *Kit* ^*WT/K641E*^. A) Widefield microscopy acquisitions. HuC/D-ir highlights soma of myenteric neurons in colon of 3-month-old WT, *Spry4* KO and *Kit* ^*WT/K641E*^ mice. B) Ratio of HuC/D-ir area in colon muscularis propria. Abbreviations: LM: longitudinal muscle layer, CM: circular muscle layer, scale bar: 100μm.
(TIF)

S17 Fig No change is length of the small intestine between genotypes. Length in cm for 3-month-old (A) and 9 month old (B) WT, *Spry4* KO and *Kit* ^*WT/K641E*^ animals.
(TIF)
