# Supplementary figures and images for: Hyperplasia of Interstitial Cells of Cajal in Sprouty Homolog 4 Deficient Mice
Source: PLoS One. 2015 Apr 29;10(4):e0124861. doi: 10.1371/journal.pone.0124861 (PMC4414615; doi:10.1371/journal.pone.0124861)

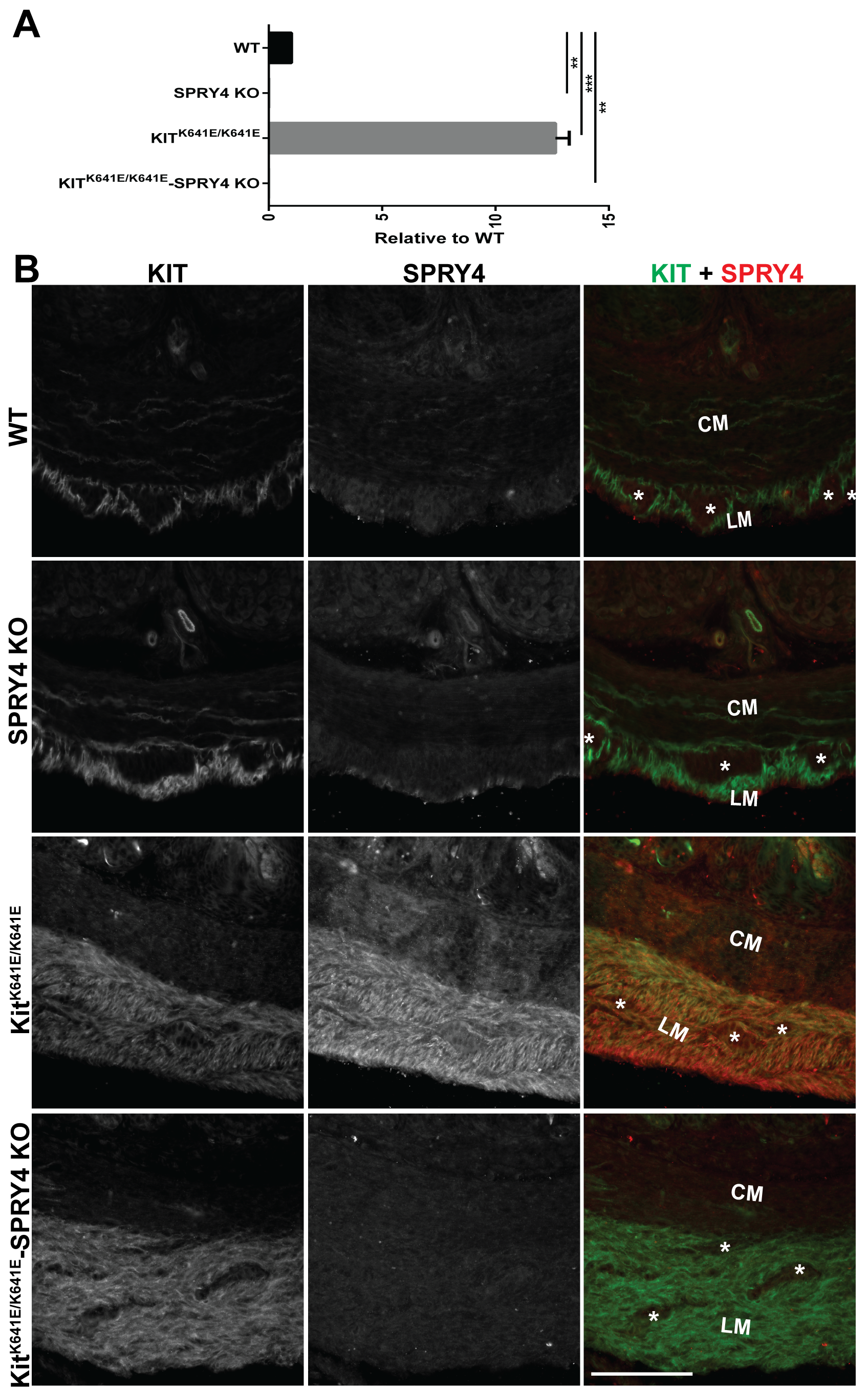

Supplement: S1 Fig — A) qPCR analysis of Spry4 mRNA levels in the postnatal (P10) mouse antrum showing a significant increase in Spry4 expression in Kit K641E/K641E antrum compared to WT, while Spry4 expression was undetectable in Spry4 KO or Kit K641E/K641E -Spry4 KO. P-values (Kruskal-Wallis test with Dunn’s post-hoc) **: p<0.01, ***: p<0.001. B) Immunofluorescence for KIT immunoreactivity (-ir) and SPRY4-ir in P10 antrum. Widefield microscopy, sequential channels acquisitions. Left column: grey scale images of KIT-ir ICC. Middle column: grey scale images of SPRY4-ir. Right column: merged images. KIT-ir and SPRY4-ir are displayed in green and in red, respectively. SPRY4-ir (red) was detected in the KIT-ir ICC (green) only in Kit K641E/K641E mice but not in WT, Spry4 KO or Kit K641E/K641E -Spry4 KO antrum. Abbreviations: LM: longitudinal muscle layer, CM: circular muscle layer, *: myenteric plexus, scale bar: 100μm. (TIF) [file pone.0124861.s001.tif]

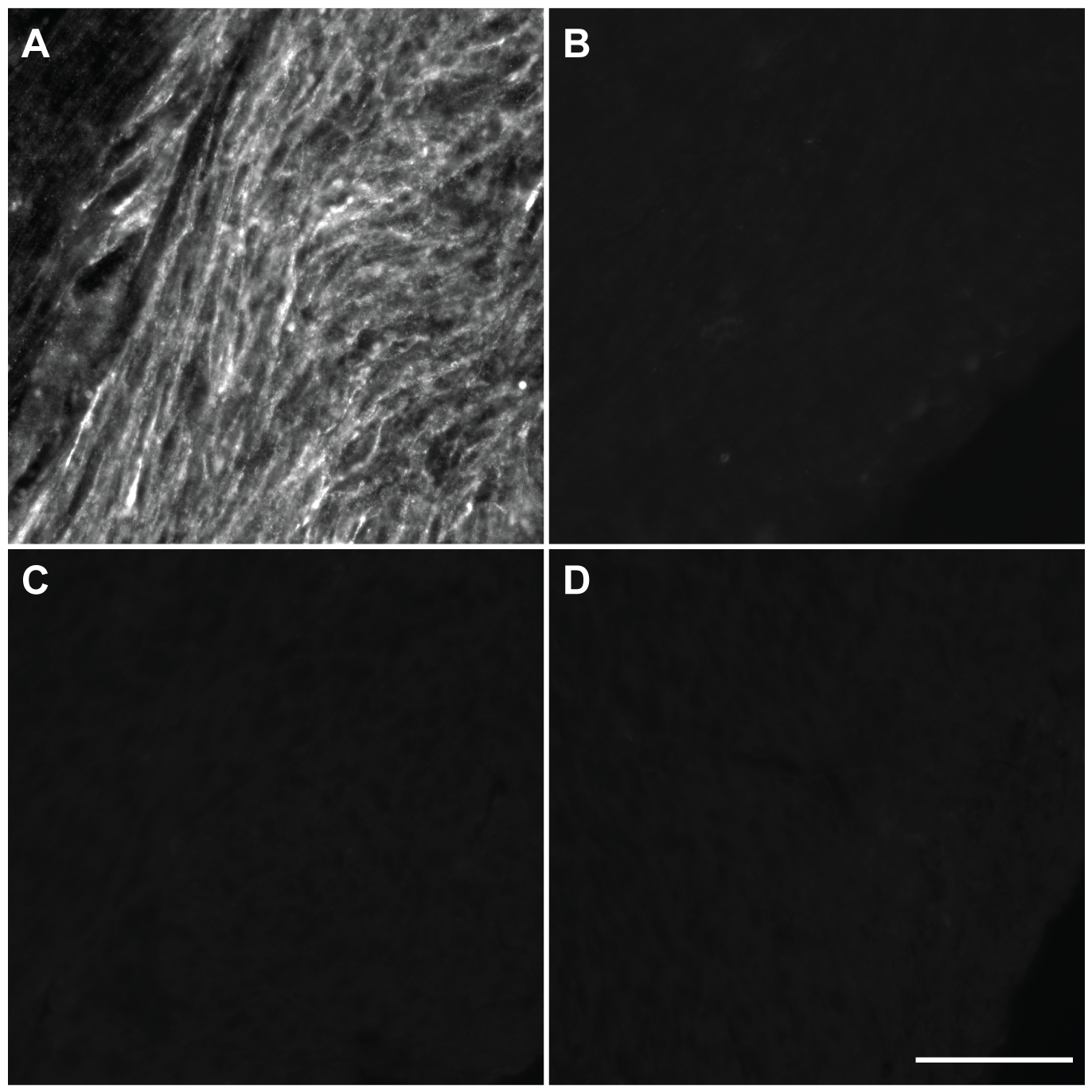

Supplement: S2 Fig — A) Control SPRY4-ir (without peptide). SPRY4 antibody preabsorption with B) 1μg or C) 0.1μg immunogenic peptide wiped out the signal. D) Negative control (omission of primary antibody). Scale bar: 50μm. (TIF) [file pone.0124861.s002.tif]

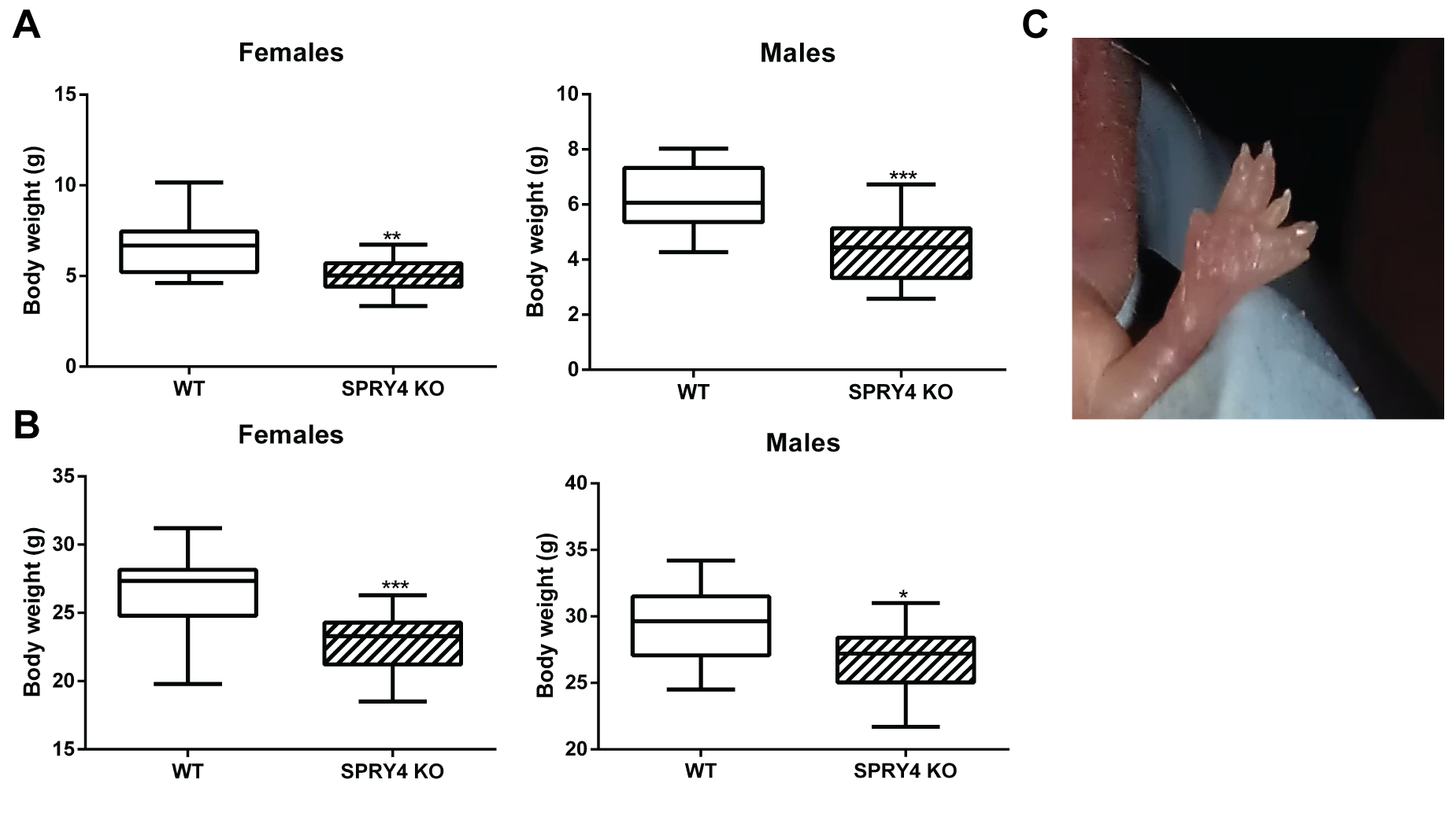

Supplement: S3 Fig — Female and male Spry4 KO animals showed a significantly lower body weight compared to their WT littermates at A) P10 and B) 3 months of age. C) An example of polysyndactyly in Spry4 KO animals. (TIF) [file pone.0124861.s003.tif]

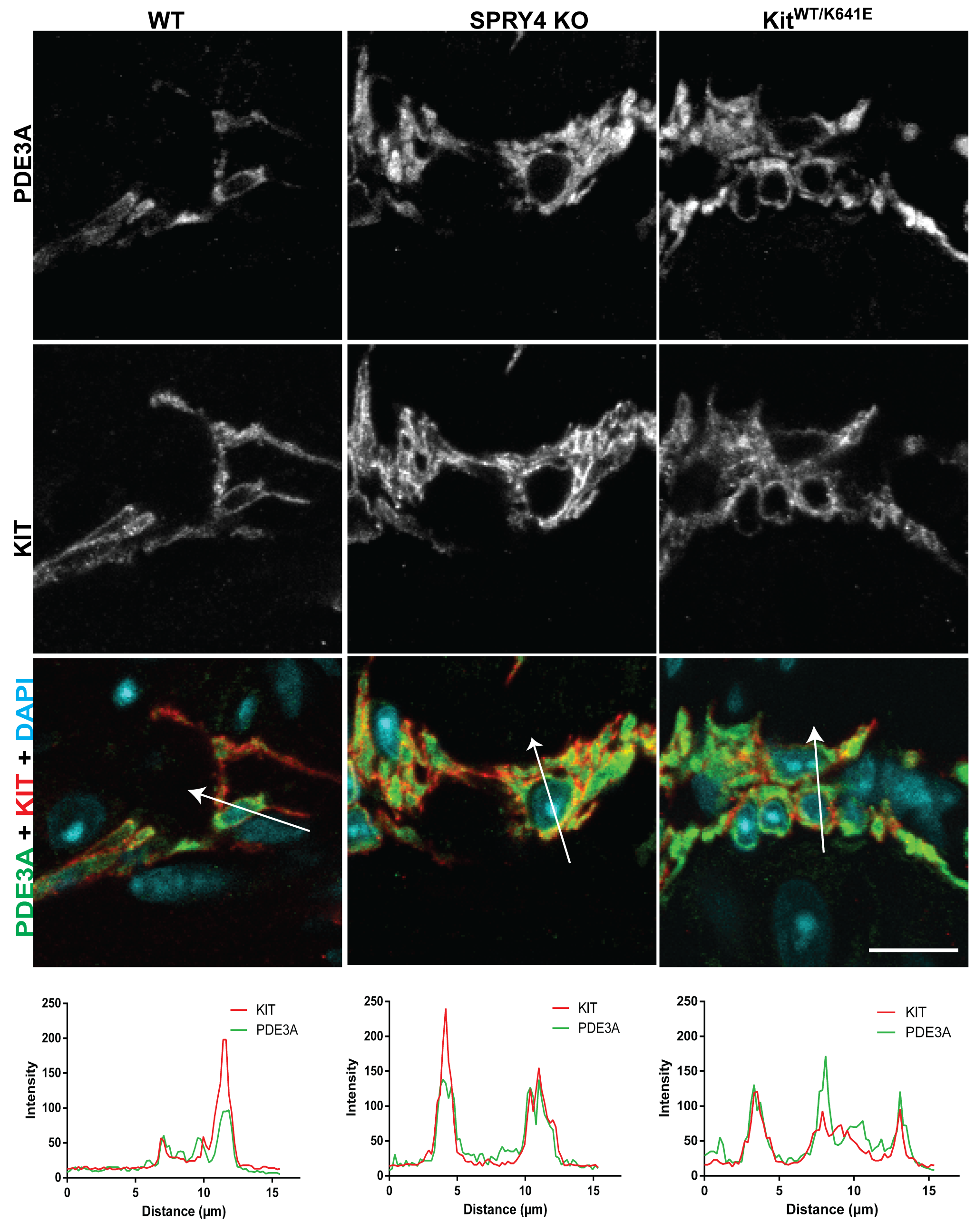

Supplement: S4 Fig — Confocal microscopy, sequential channels acquisitions. Upper row: grey scale images of PDE3A immunoreactivity (-ir) ICC. Second row: grey scale images of KIT-ir ICC. Third row: merged images. PDE3A-ir is displayed in green, KIT-ir in red, with nuclear counterstain (DAPI) in blue. Bottom row: Immunofluorescence intensity plots for PDE3A-ir and KIT-ir along the lines drawn across individual cells above, demonstrating that PDE3A-ir and KIT-r were consistently found in the same cells. Abbreviations: LM: longitudinal muscle layer, CM: circular muscle layer, scale bar: 10μm (TIF) [file pone.0124861.s004.tif]

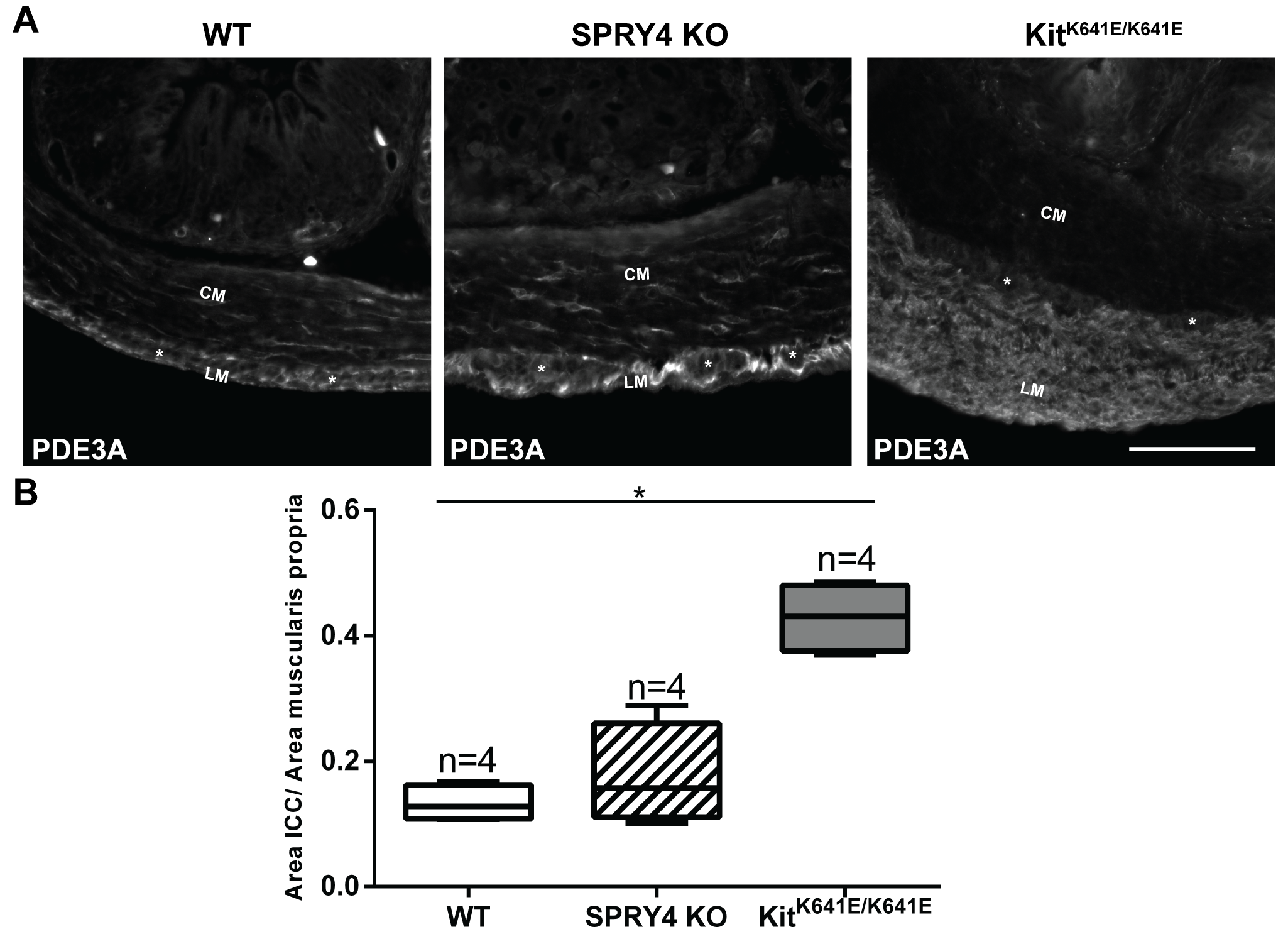

Supplement: S5 Fig — A) Widefield microscopy acquisitions. PDE3A immunoreactivity (-ir) highlights ICC in the antrum of 10 days old WT, Spry4 KO and Kit WT/K641E mice. B) Ratio of PDE3A-ir ICC area in antrum muscularis propria. Abbreviations: LM: longitudinal muscle layer, CM: circular muscle layer, *: location of myenteric plexus, scale bar: 50μm. P-values (Kruskal-Wallis with Dunn’s post hoc), *: p<0.05 (TIF) [file pone.0124861.s005.tif]

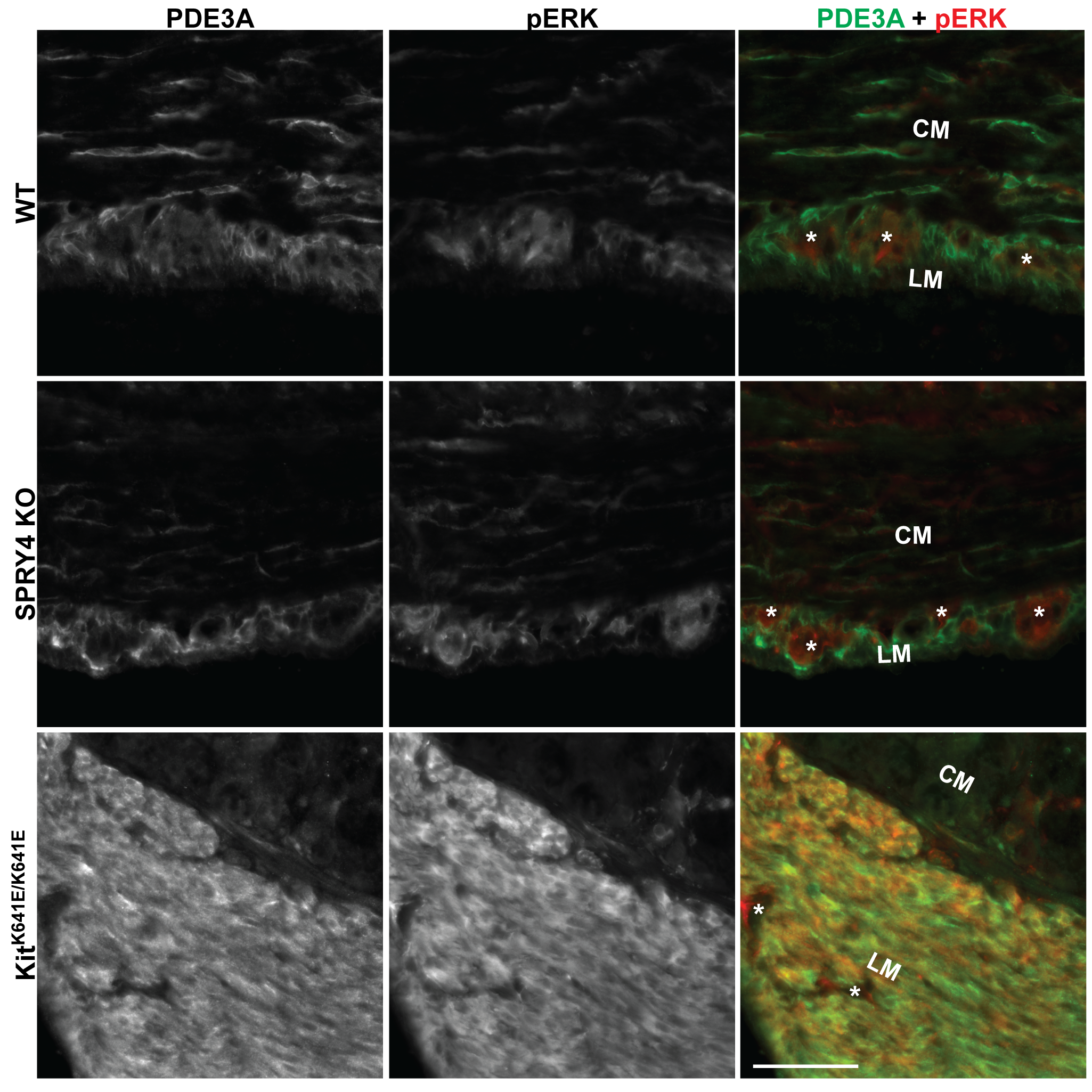

Supplement: S6 Fig — Widefield microscopy, sequential channels acquisitions. Left column: PDE3A immunoreactivity (-ir) ICC in WT, Spry4 KO and Kit WT/K641E. Middle column: pERK-ir in in the 3 genotypes. Right column: merged images: PDE3A-ir and pERK-ir displayed in green and in red, respectively. pERK-ir (red) was consistently detected in myenteric plexus and nerve fibers in the muscularis propria of all genotypes. PDE3A-ir ICC (green) which were also pERK-ir were solely detected in Kit K641E/K641E mice—and not in the other genotypes. Abbreviations: LM: longitudinal muscle layer, CM: circular muscle layer, *: location of myenteric plexus, scale bar: 50μm. (TIF) [file pone.0124861.s006.tif]

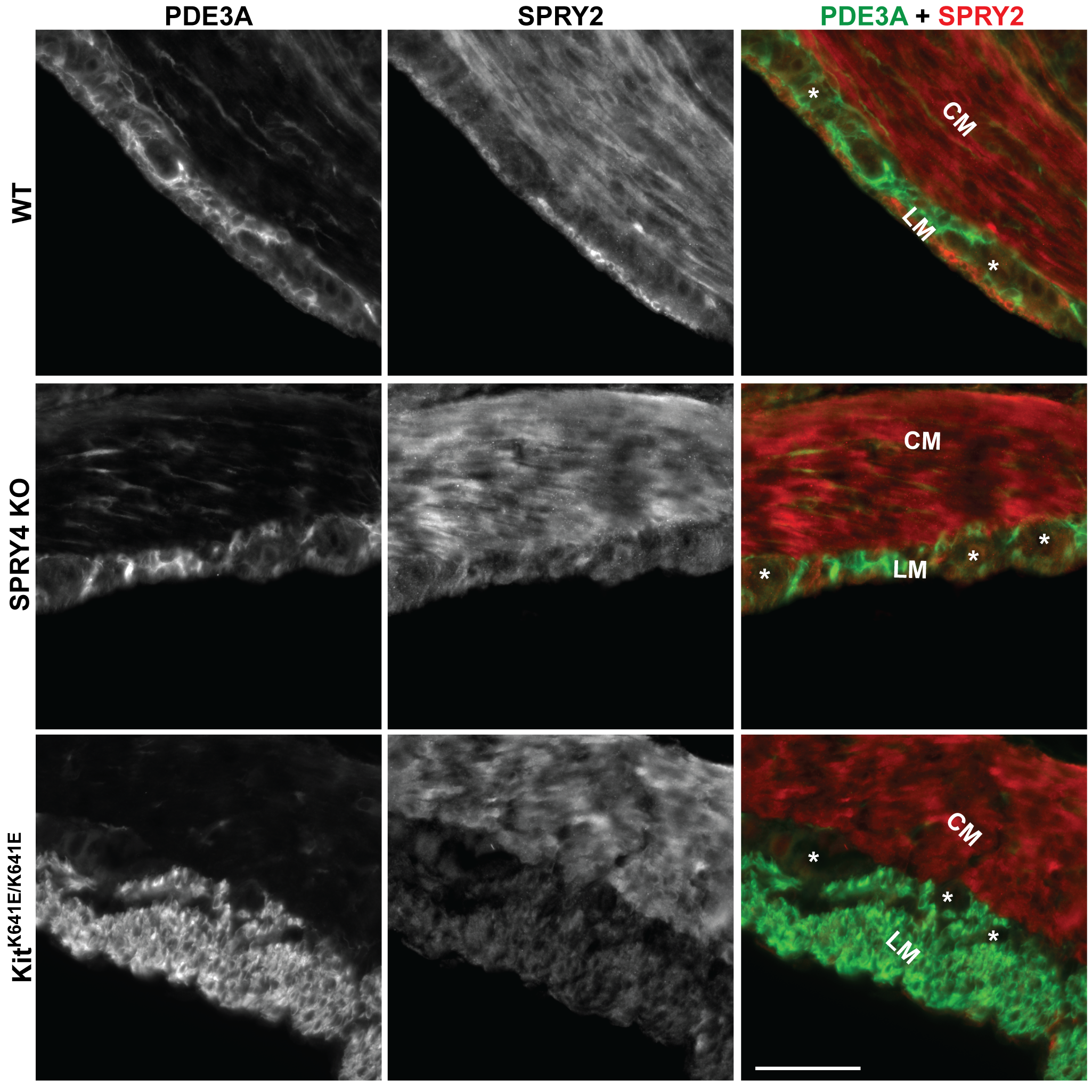

Supplement: S7 Fig — Widefield microscopy, sequential channels acquisitions. Left column: PDE3A-ir ICC in WT, Spry4 KO and Kit K641E/K641E. Middle column: SPRY2-ir in in the 3 genotypes. Right column: merged images: PDE3A-ir and SPRY2-ir displayed in green and in red, respectively. SPRY2-ir was consistently detected in smooth muscle cells of the muscularis propria but not in PDE3A-ir ICC. Abbreviations: LM: longitudinal muscle layer, CM: circular muscle layer, *: location of myenteric plexus, scale bar: 50μm. (TIF) [file pone.0124861.s007.tif]

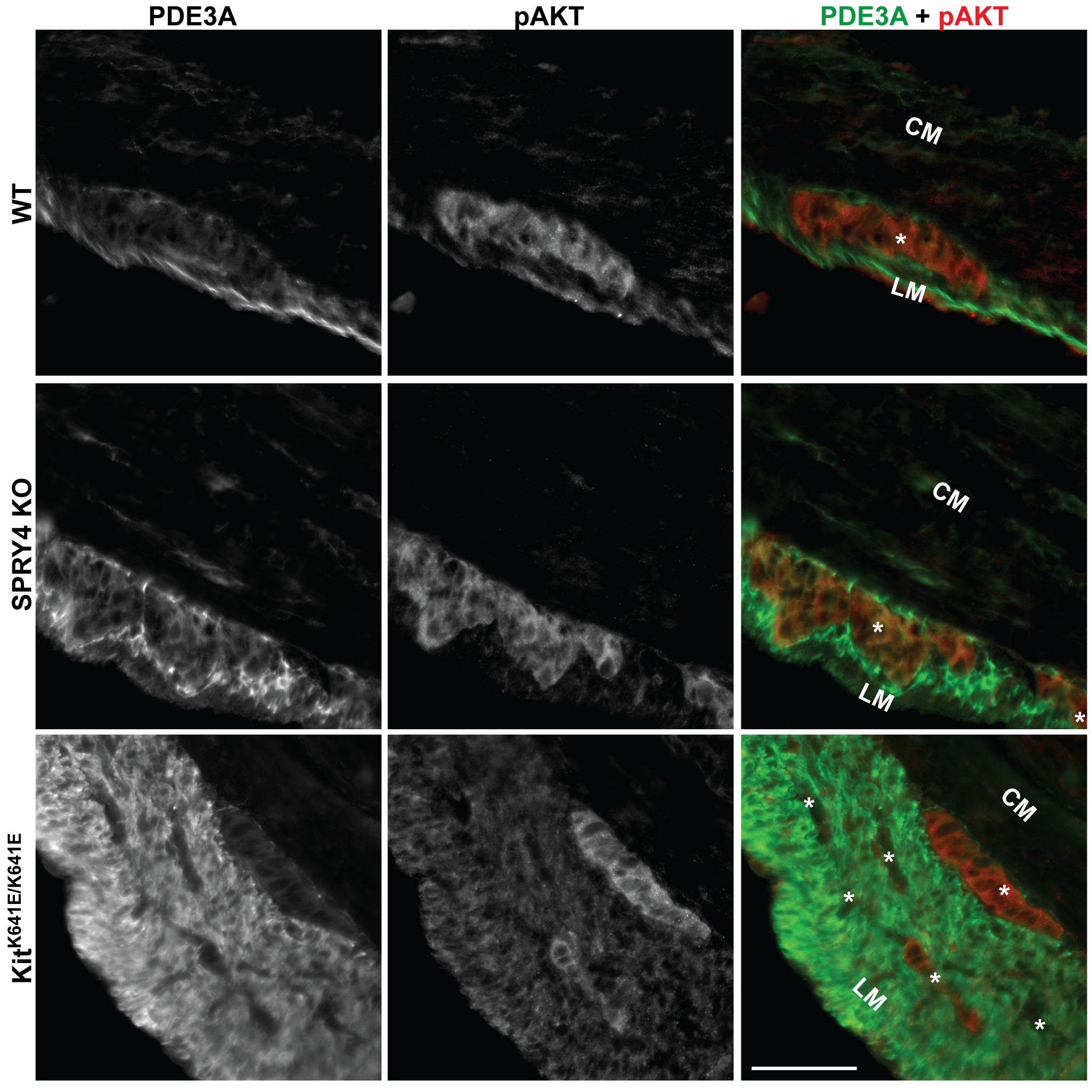

Supplement: S8 Fig — Widefield microscopy, sequential channels acquisitions. Left column: PDE3A immunoreactivity (-ir) ICC in WT, SPRY4 KO and KitWT/K641E. Middle column: pAKT-ir in in the 3 genotypes. Right column: merged images: PDE3A-ir and pAKT displayed in green and in red, respectively. pAKT (red) was consistently detected in myenteric plexus and nerve fibers in the muscularis propria but solely in PDE3A-ir ICC (green) of Kit K641E/K641E animals. Abbreviations: LM: longitudinal muscle layer, CM: circular muscle layer, *: location of myenteric plexus, scale bar: 50μm. (TIF) [file pone.0124861.s008.tif]

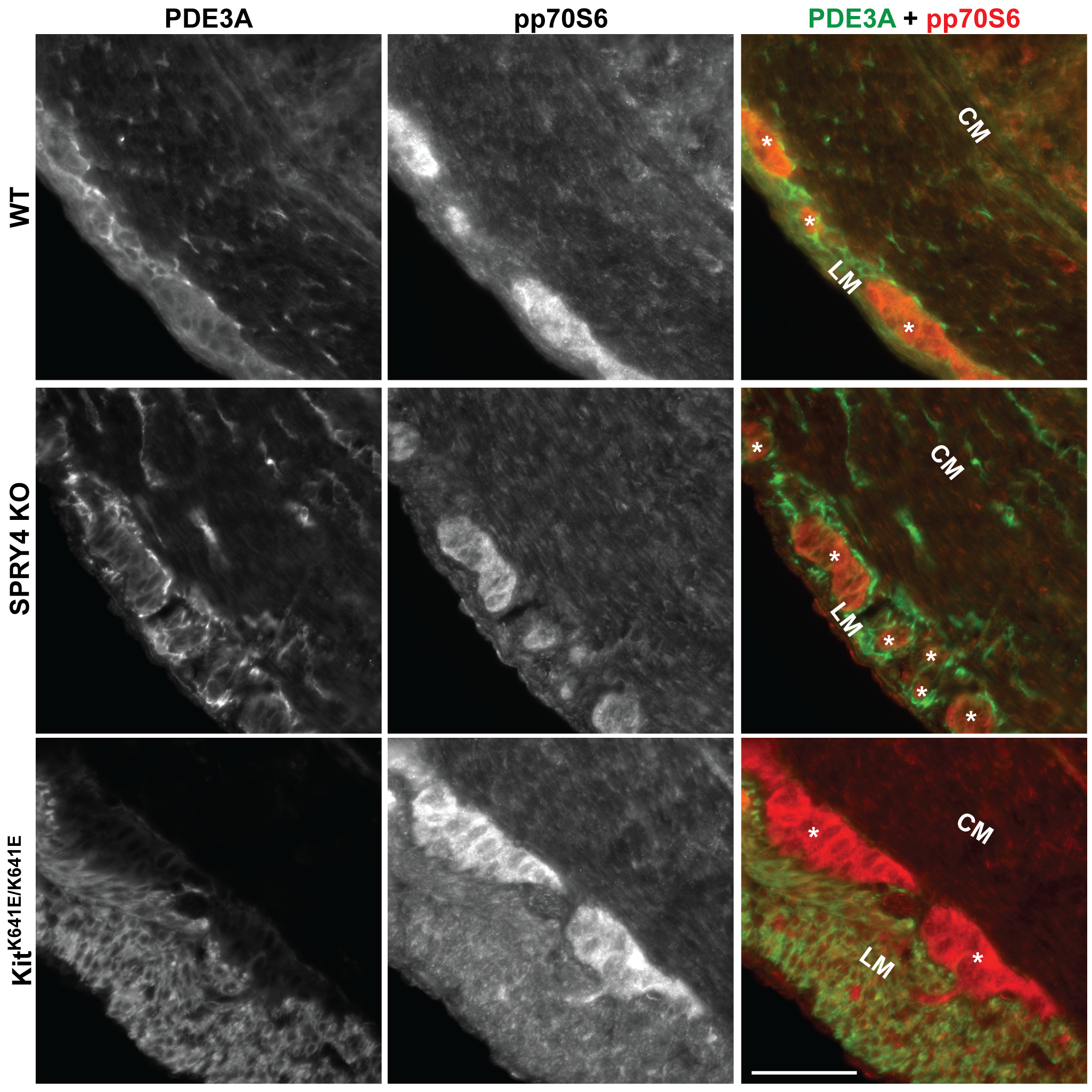

Supplement: S9 Fig — Widefield microscopy, sequential channels acquisitions.Left column: PDE3A immunoreactivity (-ir) ICC in WT, Spry4 KO and Kit K641E/K641E antrum. Middle column: pp70S6-ir for each genotype. Right column: merged images: PDE3A and pp70S6-ir displayed in green and red, respectively. pp70S6 was consistently detected in myenteric plexus and nerve fibers in the muscularis propria but solely in PDE3A-ir ICC of Kit K641E/K641E animals. Abbreviations: LM: longitudinal muscle layer, CM: circular muscle layer, *: myenteric plexus, scale bar: 50μm (TIF) [file pone.0124861.s009.tif]

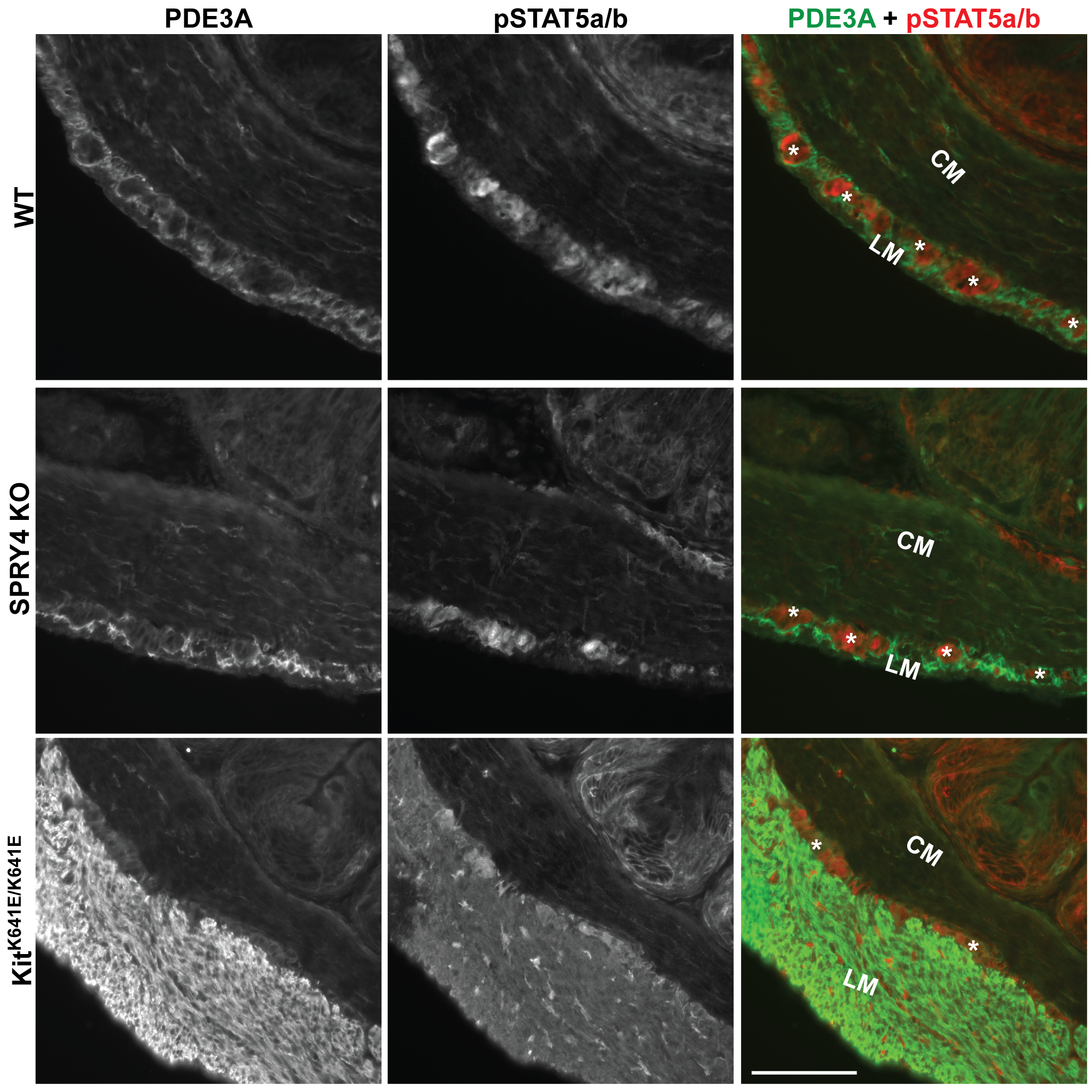

Supplement: S10 Fig — Widefield microscopy, sequential channels acquisitions. Left column: PDE3A immunoreactivity (-ir) ICC in WT, Spry4 KO and Kit K641E/K641E antrum. Middle column: pSTAT5a/b-ir for each genotype. Right column: merged images: PDE3A and pSTAT5a/b-ir displayed in green and red, respectively. pSTAT5a/b was consistently detected in myenteric plexus and nerve fibers in the muscularis propria but solely in PDE3A-ir ICC of Kit K641E/K641E animals. Abbreviations: LM: longitudinal muscle layer, CM: circular muscle layer, *: myenteric plexus, scale bar: 50μm. (TIF) [file pone.0124861.s010.tif]

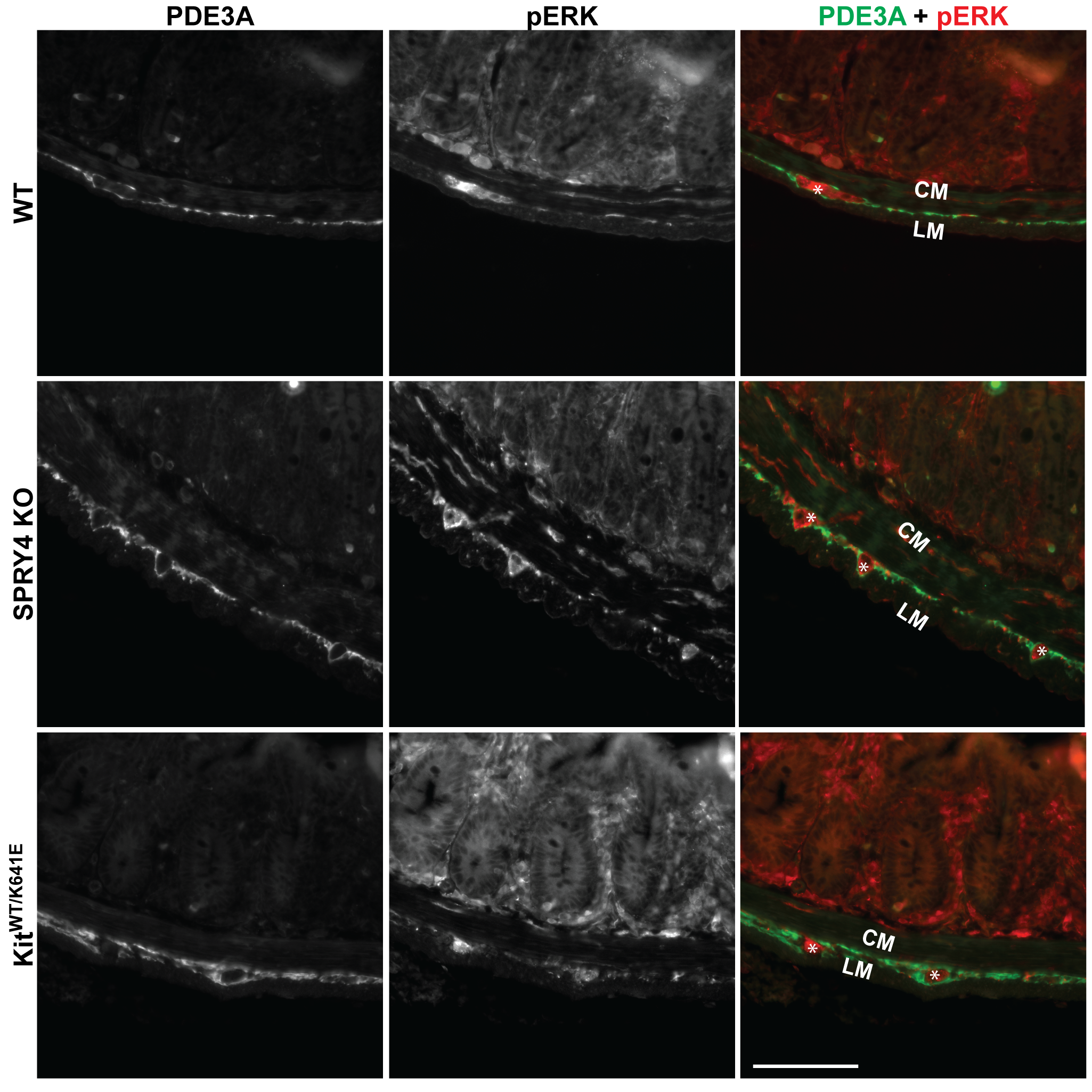

Supplement: S11 Fig — Widefield microscopy, sequential channels acquisitions.Left column: PDE3A immunoreactivity (-ir) staining ICC in WT, Spry4 KO and Kit WT/K641E. Middle column: pERK-ir in the 3 genotypes. Right column: merged images: PDE3A-ir and pERK-ir displayed in green and in red, respectively. Abbreviations: LM: longitudinal muscle layer, CM: circular muscle layer, *: location of myenteric plexus, scale bar: 100μm. (TIF) [file pone.0124861.s011.tif]

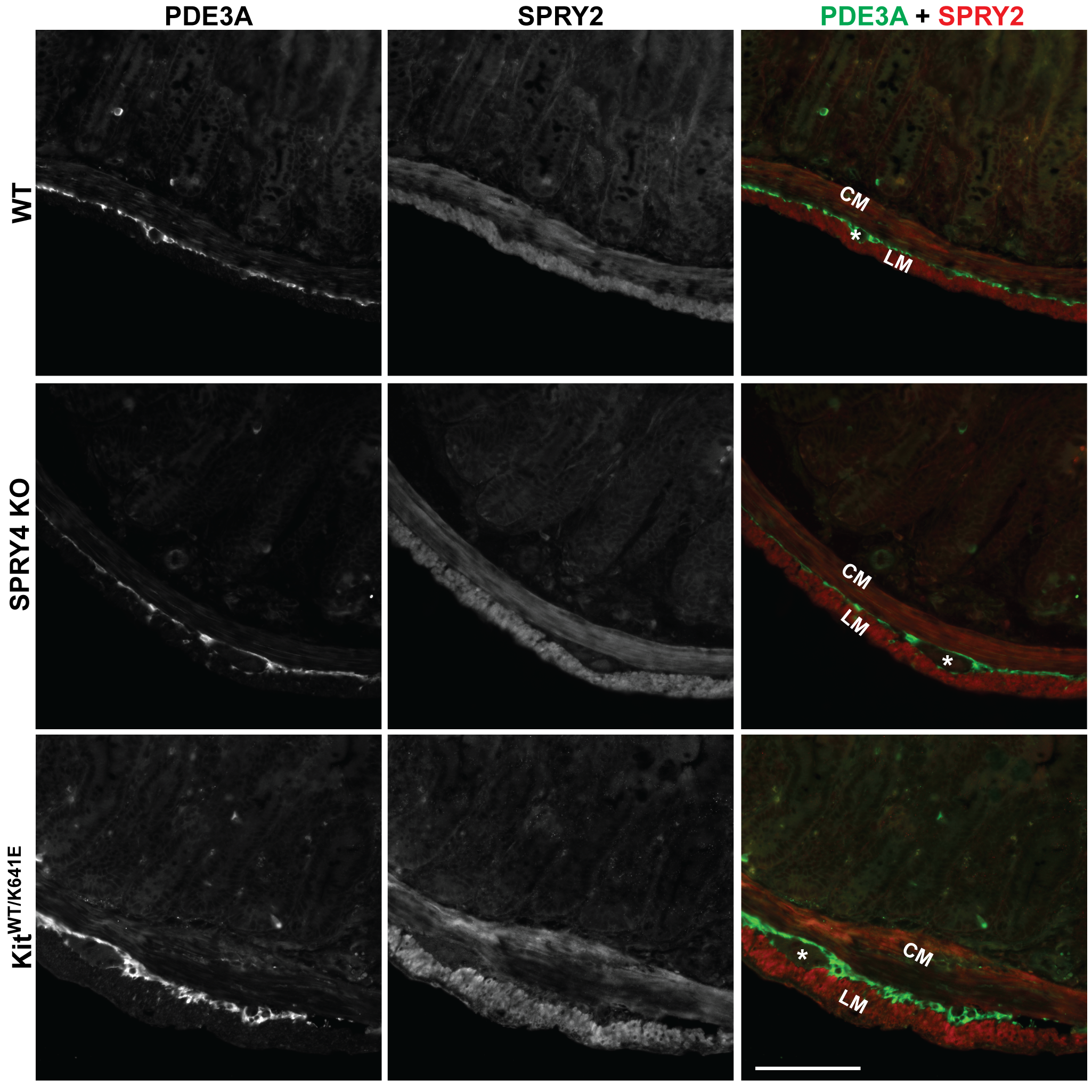

Supplement: S12 Fig — Widefield microscopy, sequential channels acquisitions.Left column: PDE3A-ir ICC in WT, Spry4 KO and Kit WT/K641E. Middle column: SPRY2-ir in in the 3 genotypes. Right column: merged images: PDE3A-ir and SPRY2-ir displayed in green and in red, respectively. SPRY2-ir (red) was consistently detected in the smooth muscle cells of the muscularis propria but not in PDE3A-ir ICC (green). Abbreviations: LM: longitudinal muscle layer, CM: circular muscle layer, *: location of myenteric plexus, scale bar: 100μm. (TIF) [file pone.0124861.s012.tif]

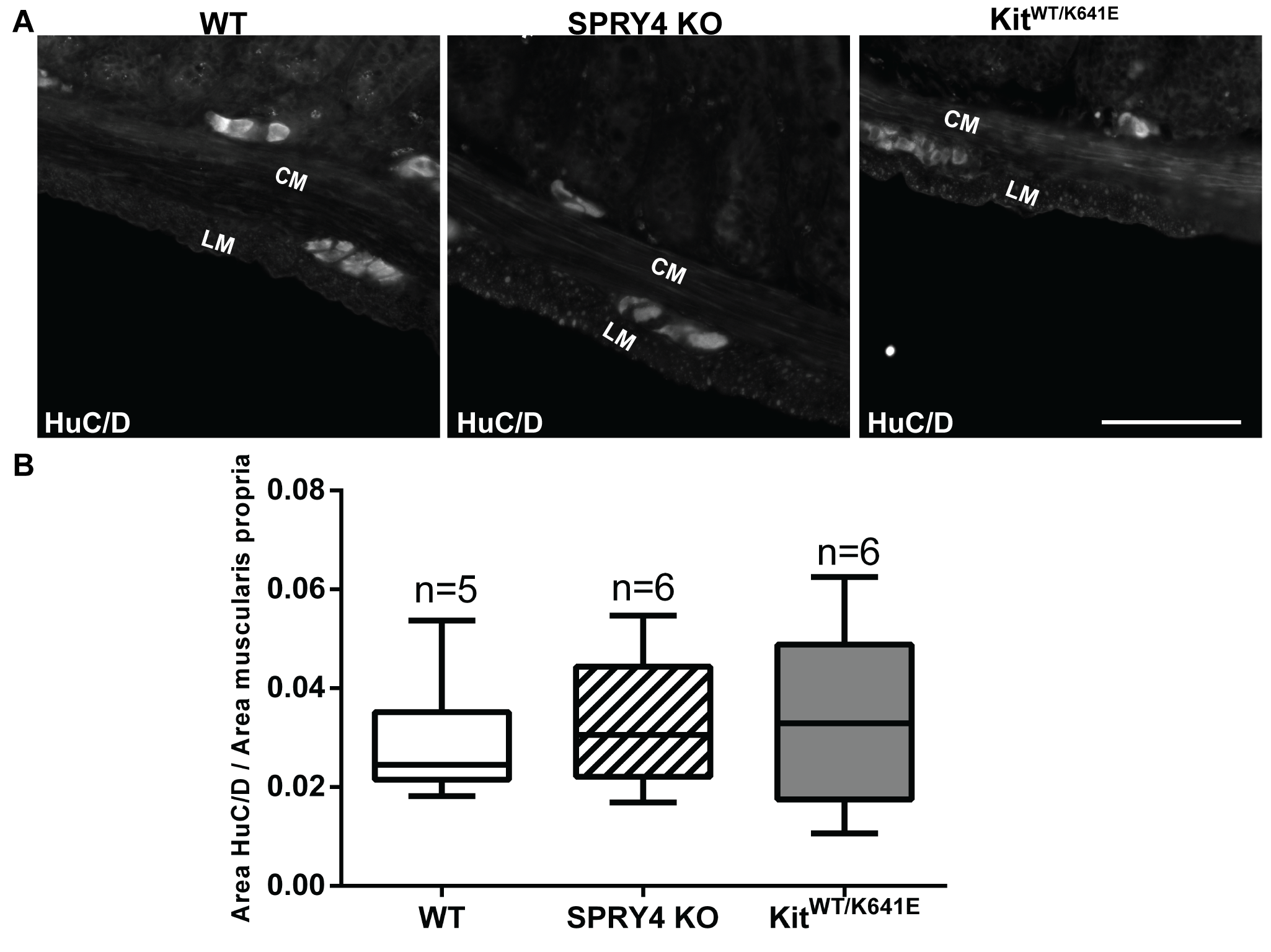

Supplement: S13 Fig — A) Widefield microscopy acquisitions. HuC/D-ir highlights the soma of myenteric neurons in small intestine of 3-month-old WT, Spry4 KO and Kit WT/K641E mice. B) Ratio of HuC/D-ir area in small intestine muscularis propria. Abbreviations: LM: longitudinal muscle layer, CM: circular muscle layer, scale bar: 100μm. (TIF) [file pone.0124861.s013.tif]

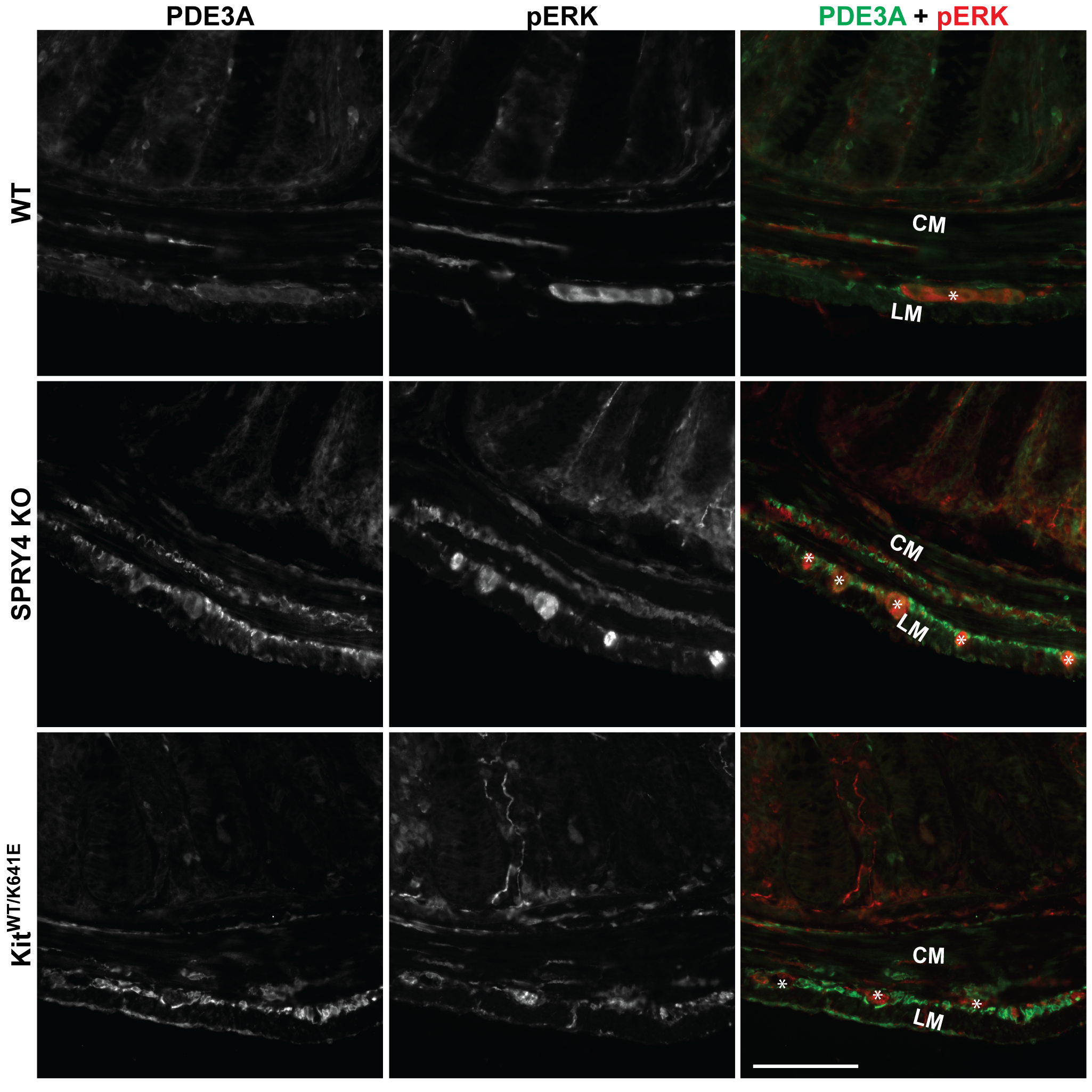

Supplement: S14 Fig — Widefield microscopy, sequential channels acquisitions.Left column: PDE3A immunoreactivity (-ir) staining ICC in WT, Spry4 KO and Kit WT/K641E. Middle column: pERK-ir in the 3 genotypes. Right column: merged images: PDE3A-ir and pERK-ir displayed in green and in red, respectively. Abbreviations: LM: longitudinal muscle layer, CM: circular muscle layer, *: location of myenteric plexus, scale bar: 100μm. (TIF) [file pone.0124861.s014.tif]

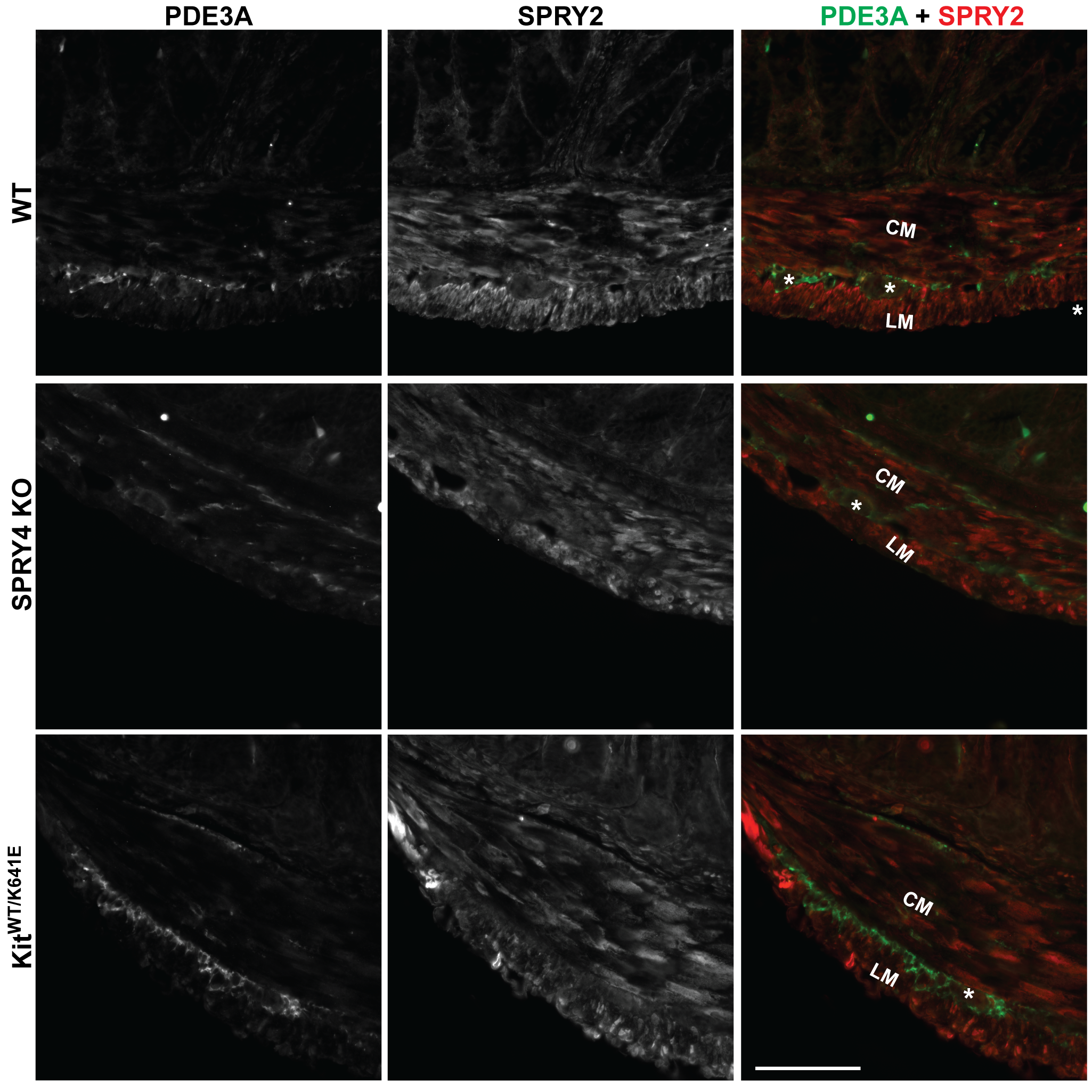

Supplement: S15 Fig — Widefield microscopy, sequential channels acquisitions.Left column: PDE3A-ir ICC in WT, Spry4 KO and Kit WT/K641E. Middle column: SPRY2-ir in in the 3 genotypes. Right column: merged images: PDE3A-ir and SPRY2-ir displayed in green and in red, respectively. SPRY2-ir (red) was consistently detected in the smooth muscle cells of the muscularis propria but not in PDE3A-ir ICC (green). Abbreviations: LM: longitudinal muscle layer, CM: circular muscle layer, *: location of myenteric plexus, scale bar: 100μm. (TIF) [file pone.0124861.s015.tif]

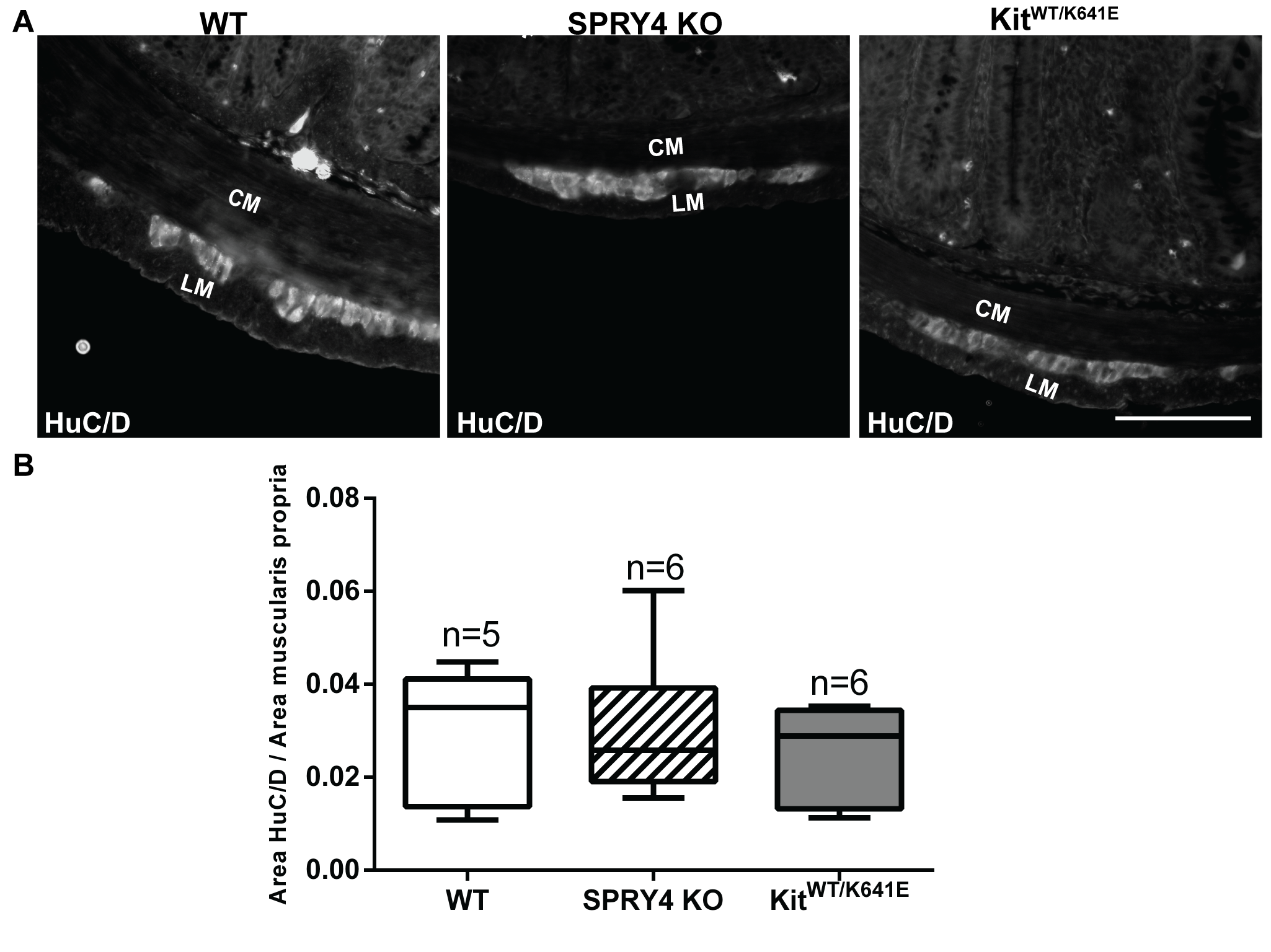

Supplement: S16 Fig — A) Widefield microscopy acquisitions. HuC/D-ir highlights soma of myenteric neurons in colon of 3-month-old WT, Spry4 KO and Kit WT/K641E mice. B) Ratio of HuC/D-ir area in colon muscularis propria. Abbreviations: LM: longitudinal muscle layer, CM: circular muscle layer, scale bar: 100μm. (TIF) [file pone.0124861.s016.tif]

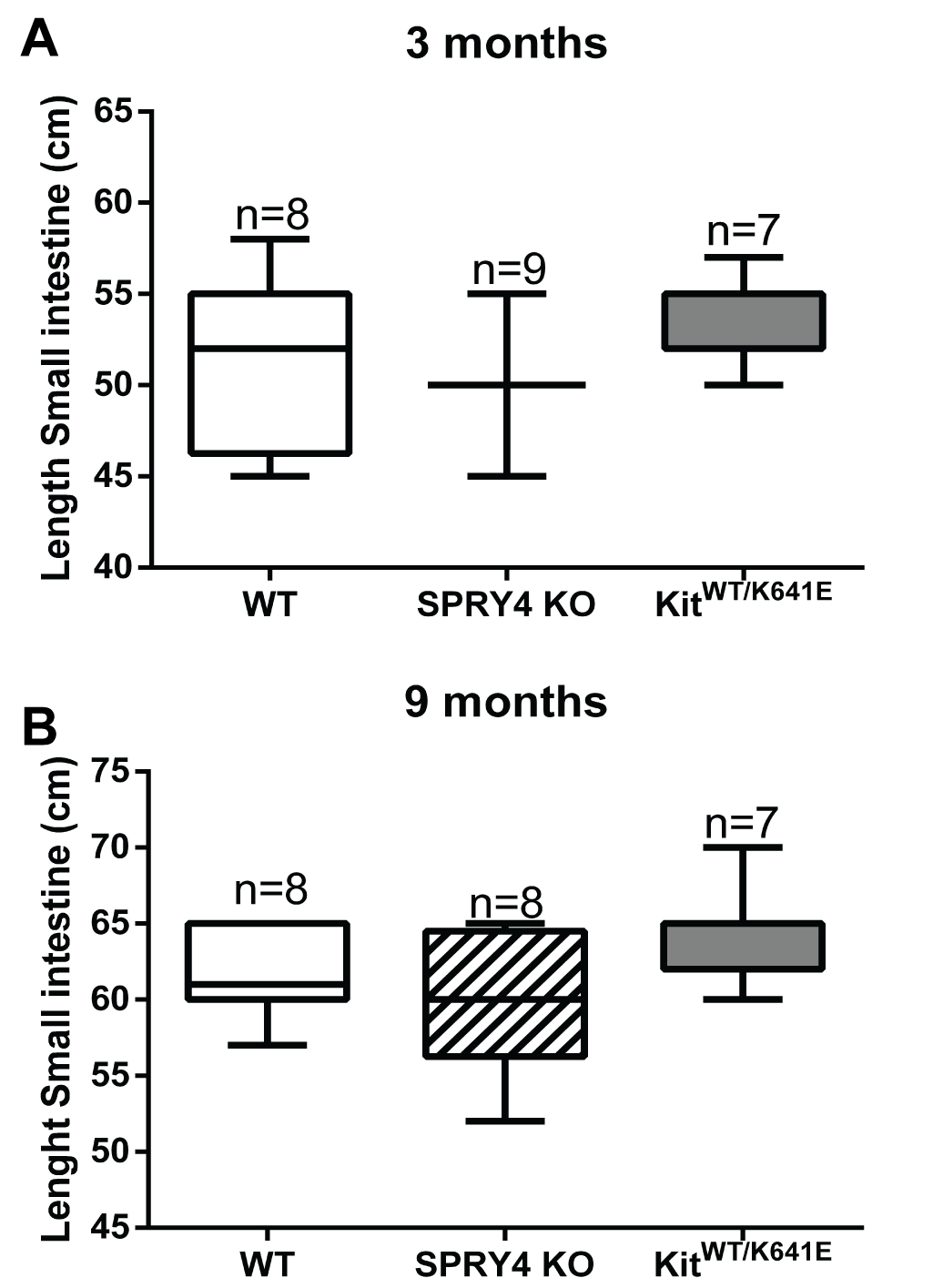

Supplement: S17 Fig — Length in cm for 3-month-old (A) and 9 month old (B) WT, Spry4 KO and Kit WT/K641E animals. (TIF) [file pone.0124861.s017.tif]
